# The HIF-prolyl hydroxylases have distinct and nonredundant roles in colitis-associated cancer

**DOI:** 10.1172/jci.insight.153337

**Published:** 2022-11-22

**Authors:** Kilian B. Kennel, Julius Burmeister, Praveen Radhakrishnan, Nathalia A. Giese, Thomas Giese, Martin Salfenmoser, Jasper M. Gebhardt, Moritz J. Strowitzki, Cormac T. Taylor, Ben Wielockx, Martin Schneider, Jonathan M. Harnoss

**Affiliations:** 1 Department of General, Visceral and Transplantation Surgery and; 2 Institute of Immunology, University Hospital Heidelberg, Heidelberg, Germany.; 3 School of Medicine, Systems Biology Ireland, and the Conway Institute of Biomolecular and Biomedical Research, University College Dublin, Dublin, Ireland.; 4 Institute for Clinical Chemistry and Laboratory Medicine, Dresden University of Technology, Dresden, Germany.

**Keywords:** Inflammation, Oncology, Colorectal cancer, Hypoxia, Inflammatory bowel disease

## Abstract

Colitis-associated colorectal cancer (CAC) is a severe complication of inflammatory bowel disease (IBD). HIF-prolyl hydroxylases (PHD1, PHD2, and PHD3) control cellular adaptation to hypoxia and are considered promising therapeutic targets in IBD. However, their relevance in the pathogenesis of CAC remains elusive. We induced CAC in *Phd1^–/–^*, *Phd2^+/–^*, *Phd3^–/–^*, and WT mice with azoxymethane (AOM) and dextran sodium sulfate (DSS). *Phd1^–/–^* mice were protected against chronic colitis and displayed diminished CAC growth compared with WT mice. In *Phd3^–/–^* mice, colitis activity and CAC growth remained unaltered. In *Phd2^+/–^* mice, colitis activity was unaffected, but CAC growth was aggravated. Mechanistically, *Phd2* deficiency (i) increased the number of tumor-associated macrophages in AOM/DSS-induced tumors, (ii) promoted the expression of EGFR ligand epiregulin in macrophages, and (iii) augmented the signal transducer and activator of transcription 3 and extracellular signal–regulated kinase 1/2 signaling, which at least in part contributed to aggravated tumor cell proliferation in colitis-associated tumors. Consistently, *Phd2* deficiency in hematopoietic (*Vav:Cre-Phd2^fl/fl^*) but not in intestinal epithelial cells (*Villin:Cre-Phd2^fl/fl^*) increased CAC growth. In conclusion, the 3 different PHD isoenzymes have distinct and nonredundant effects, promoting (PHD1), diminishing (PHD2), or neutral (PHD3), on CAC growth.

## Graphical abstract

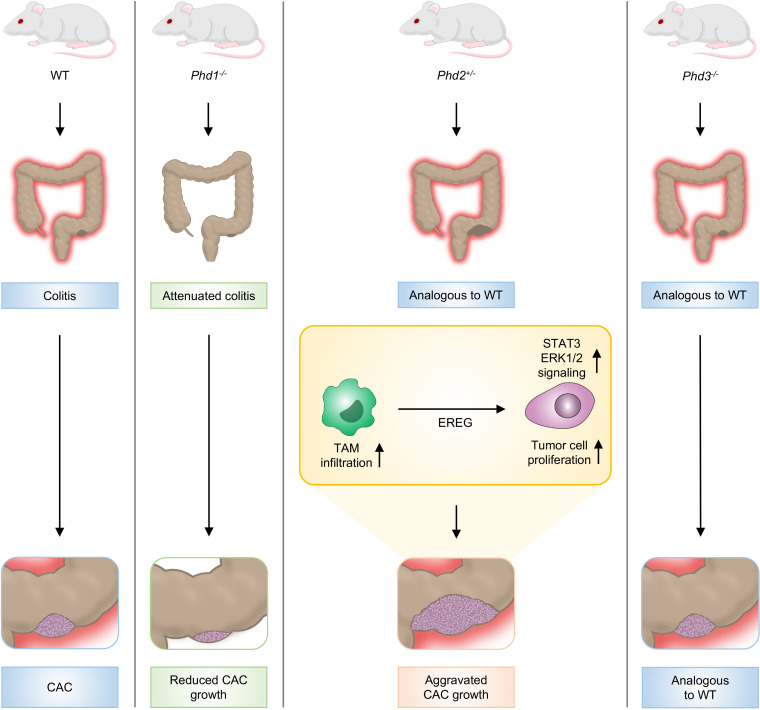

## Introduction

Inflammatory bowel disease (IBD), including ulcerative colitis (UC) and Crohn’s disease (CD) (1, 2), is associated with a significantly increased risk for colitis-associated colorectal cancer (CAC). The severity, extent, and duration of inflammation are the key risk factors for CAC (3, 4). Compared with sporadic colorectal cancer (CRC), CAC occurs earlier and is associated with significant morbidity and mortality (5, 6). While antiinflammatory therapies are the mainstay of current IBD treatment regimens (7), medically refractory UC and occurrence of CAC are the main indications for colectomy (8).

In the search for novel therapies for IBD, the HIF-prolyl hydroxylases (PHD1, PHD2, and PHD3) have emerged as putative therapeutic targets (9). The PHD isoenzymes are molecular oxygen sensors orchestrating cellular adaptation to hypoxia by regulating the stability of HIF (10). In normoxia, PHDs hydroxylate the HIFα subunit, thereby promoting its degradation via the ubiquitin proteasome system. In hypoxia, PHD-mediated oxygen-dependent hydroxylation of the HIFα subunit is inhibited and HIF is thus stabilized. HIF initiates various transcriptional programs to adapt cells to hypoxia: angiogenesis and erythropoiesis to increase oxygen supply or adaptation of cellular metabolism to decrease oxygen consumption (11).

In IBD, the intestinal epithelium is hypoxic, a condition termed “inflammatory hypoxia” (12). Accordingly, the expression of HIF and specifically PHD1 is increased in experimentally induced colitis in mice and colon biopsies from patients with UC and CD (13, 14). Strikingly, genetic loss of *Phd1* or small-molecule pan-PHD inhibition attenuates disease activity in various rodent IBD models (15–19). In cancer and particularly in CRC, the PHD isoenzymes have divergent roles. While the expression of PHD1 inhibits tumor growth in a CRC xenograft model (20), PHD3 deficiency in human CRC biopsies is associated with tumor progression and poor clinical outcome (21, 22). For PHD2, both tumor-promoting and tumor-suppressive functions have been reported (23, 24). Due to these heterogeneous biological functions of the 3 PHD isoenzymes in IBD and CRC, their relevance in the pathogenesis of CAC remains elusive.

Here, we investigated the importance of PHD1–3 in an azoxymethane- (AOM-) and dextran sodium sulfate–induced (DSS-induced) mouse model of CAC. Moreover, to gain a deeper understanding of the specific role of PHD2 — the key oxygen sensor (25) — in the pathogenesis of CAC, we used transgenic mouse models harboring a tissue-specific deletion of *Phd2*. We demonstrate that the 3 PHD isoenzymes have distinct, whether promoting (PHD1), diminishing (PHD2), or neutral (PHD3), effects on CAC growth in the AOM/DSS model. Mechanistically, mitigation of CAC growth by *Phd2* is at least in part mediated by expression of *Phd2* in tumor-associated macrophages (TAMs). *Phd2* reduces the number of TAMs and expression of protumorigenic epiregulin (*Ereg*) in both BM-derived macrophages (BMDMs) and AOM/DSS tumors, and, thus, at least in part contributes to the suppression of the oncogenic signal transducer and activator of transcription 3 (STAT3) and extracellular signal–regulated kinase 1/2 (ERK1/2) signaling pathways in the AOM/DSS model.

## Results

### Loss of Phd1 but not Phd2 or Phd3 selectively protects mice against chronic colitis.

**Figure 1:**
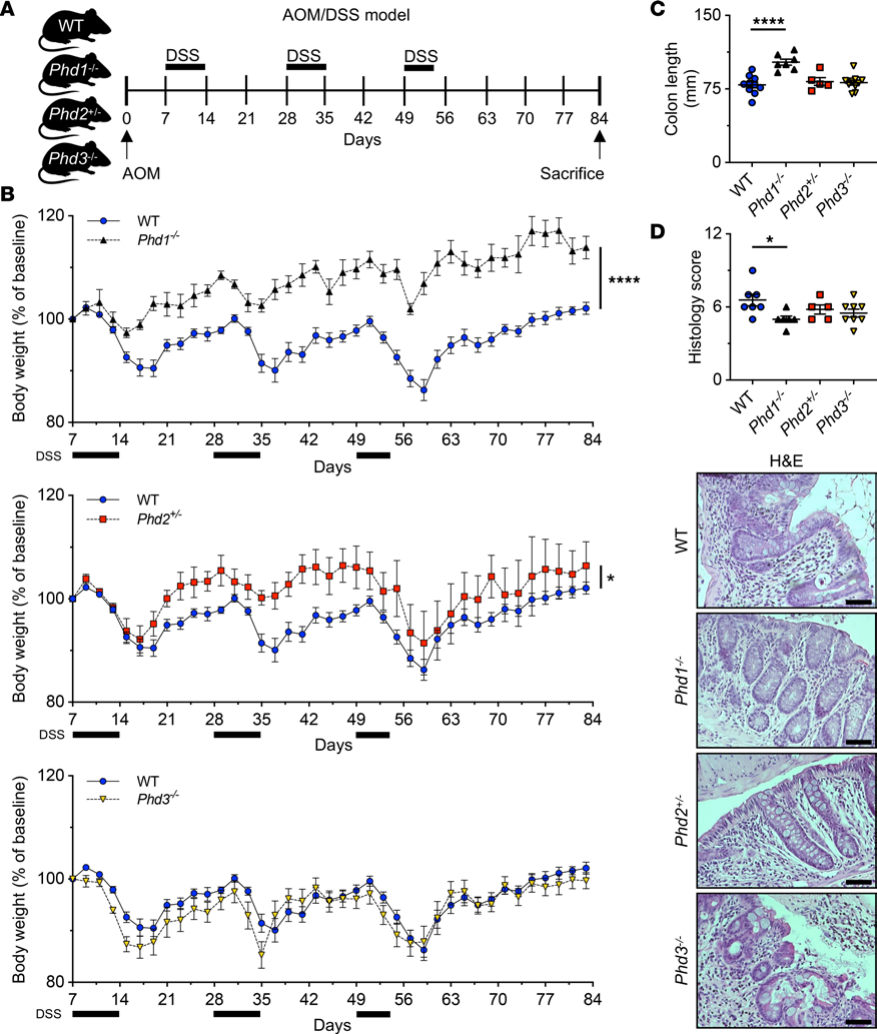
Loss of *Phd1* but not *Phd2* or *Phd3* selectively protects mice against chronic colitis. (**A**) Model of chronic colitis and colitis-associated tumorigenesis induced by AOM and repeated cycles of DSS in WT control, *Phd1^–/–^*, *Phd2^+/–^*, and *Phd3^–/–^* mice. (**B**) BW change relative to baseline from WT (*n* = 10), *Phd1^–/–^* (*n* = 6), *Phd2^+/–^* (*n* = 5), and *Phd3^–/–^* (*n* = 9) mice over the course of AOM/DSS treatment. BW was measured every other day. (**C**) Colon length of AOM/DSS-treated WT (*n* = 10), *Phd1^–/–^* (*n* = 7), *Phd2^+/–^* (*n* = 5), and *Phd3^–/–^* (*n* = 10) mice after termination of the experiment at day 84. (**D**) Histological scoring of mucosal damage (*top*) as previously described by Katakura et al. (62) and representative H&E staining (*bottom*) of colons from WT (*n* = 7), *Phd1^–/–^* (*n* = 6), *Phd2^+/–^* (*n* = 5), and *Phd3^–/–^* (*n* = 8) mice after termination of the experiment on day 84. Scale bar: 100 μm. Statistical significance was calculated using 2-way ANOVA (**B**) or 1-way ANOVA with Dunnett’s multiple comparisons test in **C** and **D**. *P < 0.05, ****P < 0.0001.

Several studies have shown that loss of *Phd1* is protective against acute colitis (15, 16). However, the importance of PHD1–3 in colitis-associated colon carcinogenesis is not yet known. To assess the putative functions of PHD1–3 in CAC, we employed the AOM/DSS model (26) in *Phd1-*, *Phd2-*, or *Phd3-*deficient (*Phd1^–/–^*, *Phd2^+/–^*, and *Phd3^–/–^*) mice and WT controls; after AOM-induced epithelial mutagenesis, mice underwent repeated cycles of DSS exposure followed by a recovery period (Figure 1A). In this chronic colitis model, *Phd1^–/–^* mice showed significantly attenuated colitis activity compared with WT control animals as assessed by the BW change and disease activity index (DAI) (Figure 1B, and Supplemental Figure 1A; supplemental material available online with this article; https://doi.org/10.1172/jci.insight.153337DS1). *Phd2^+/–^* mice showed a significantly higher BW compared with WT control mice. However, this change was less pronounced compared with *Phd1^–/–^* mice and not reflected by the more comprehensive DAI (Figure 1B, and Supplemental Figure 1A). *Phd3*^–/–^ mice showed an unchanged BW and DAI compared with WT control mice (Figure 1B, and Supplemental Figure 1A). In keeping with this, colitis-induced shortening of the colon was reduced in *Phd1^–/–^* but not *Phd2^+/–^* or *Phd3^–/–^* mice compared with WT controls (Figure 1C). Histological assessment of colonic mucosa after chronic DSS-induced colitis by H&E staining (parameters outlined in Supplemental Table 1) revealed decreased histological injury of *Phd1^–/–^* mice compared with WT control mice (Figure 1D). To further characterize the extent of inflammation, we assessed the expression of several proinflammatory cytokine and chemokine transcripts in colonic mucosa samples by semiquantitative real-time PCR (qRT-PCR). However, expression of none of the proinflammatory cytokines and chemokines was significantly changed in *Phd1^–/–^*, *Phd2*^+/–^, or *Phd3^–/–^* mice compared with their WT counterparts (Supplemental Figure 1B).

Taken together, these results demonstrate that loss of *Phd1*, but not *Phd2* or *Phd3*, is protective against DSS-induced chronic colitis.

### Loss of Phd1 diminishes, Phd2 haplodeficiency aggravates, and Phd3 deficiency does not affect colitis-associated tumor growth.

**Figure 2:**
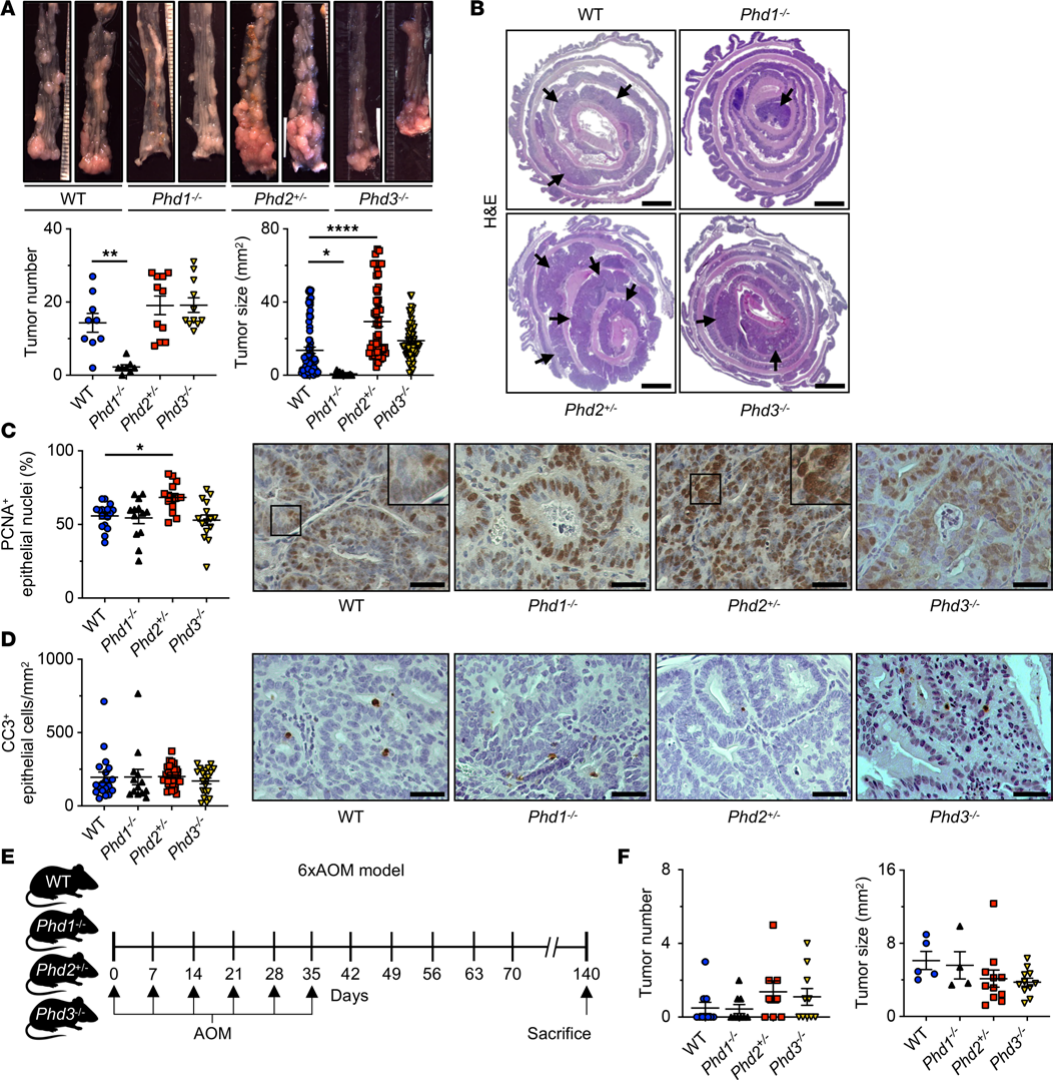
Loss of *Phd1* diminishes, *Phd2* haplodeficiency aggravates, and *Phd3* deficiency does not alter colitis-associated tumor growth. (**A**) Representative macroscopic images (*top*) and macroscopic quantification of AOM/DSS-induced tumors (*bottom*). Number of tumors per mouse (*left*; WT: *n* = 9; *Phd1^-/-^*: *n* = 9; *Phd2^+/-^*: *n* = 11; and *Phd3^-/-^*: *n* = 11 mice) and size of individual tumors (*right*; WT: *n* = 80; *Phd1^-/-^*: *n* = 12; *Phd2^+/-^*: *n* = 54; and *Phd3^-/-^*: *n* = 67 tumors). (**B**) H&E staining of colons from WT control, *Phd1^–/–^*, *Phd2^+/–^*, and *Phd3^–/–^* mice. Arrows indicate colitis-associated tumors. Scale bar: 2 mm. (**C**) Quantification of epithelial PCNA immunostaining in AOM/DSS-induced tumors (WT: *n* = 17; *Phd1^-/-^*: *n* = 13; *Phd2^+/-^*: *n* = 14; and *Phd3^-/-^*: *n* = 15 tumors) and representative histological images (*right*). Scale bar: 25 μm. (**D**) Quantification of epithelial CC3 immunostaining in AOM/DSS-induced tumors (WT: *n* = 19; *Phd1^–/–^*: *n* = 13; *Phd2^+/–^*: *n* = 32; and *Phd3^–/–^*: *n* = 20 tumors) and representative histological images (*right*). Scale bar: 25 μm. (**E**) Model of sporadic colorectal carcinogenesis induced by repeated injections of AOM (6xAOM) in WT control, *Phd1^–/–^*, *Phd2^+/–^*, and *Phd3^–/–^* mice. (**F**) Macroscopic quantification of 6xAOM-induced tumors. Number of tumors per mouse (*left*; WT: *n* = 10; *Phd1^–/–^*: *n* = 9; *Phd2^+/–^*: *n* = 8; and *Phd3^–/–^*: *n* = 10 mice) and size of individual tumors (*right*; WT: *n* = 5; *Phd1^–/–^*: *n* = 4; *Phd2^+/–^*: *n* = 11; and *Phd3^–/–^*: *n* = 11 tumors). Statistical significance was calculated using 1-way ANOVA with Dunnett’s multiple comparisons test in **A**, **C**, **D**, and **F**. *P < 0.05, **P < 0.01, ****P < 0.0001.

To evaluate the effect of *Phd1*, *2*, or *3* deficiency on colitis-associated tumorigenesis, we analyzed tumor formation and size after AOM/DSS-induced CAC. Consistent with the protective effects of *Phd1* deficiency against chronic colitis, *Phd1^–/–^* mice displayed a significantly reduced tumor number and size compared with WT controls (Figure 2, A and B, and Supplemental Figure 2A). Strikingly, although the number of tumors in *Phd2^+/–^* mice was unchanged compared with control mice, the tumors in *Phd2^+/–^* animals were significantly larger (Figure 2, A and B, and Supplemental Figure 2A). In *Phd3^–/–^* mice, tumor number and size did not differ from WT mice (Figure 2, A and B, and Supplemental Figure 2A). This indicates that the PHD isoenzymes PHD1–3 each has a distinct impact on tumor formation and growth.

To further investigate tumor cell proliferation and apoptosis in these tumors, we performed IHC of proliferating cell nuclear antigen (PCNA) and cleaved caspase-3 (CC3). Remarkably, this revealed that, while tumor proliferation was unchanged in *Phd1^–/–^* and *Phd3^–/–^* tumors compared with control animals, *Phd2^+/–^* tumors proliferated significantly more (Figure 2C). Cell apoptosis in the tumors was unchanged among all experimental groups (Figure 2D). Collectively, these results demonstrate that loss of *Phd1* diminishes CAC growth, whereas *Phd2* haplodeficiency increases tumor proliferation and, thus, colitis-associated tumor growth. Loss of *Phd3* did not result in any changes in tumor burden in the AOM/DSS model.

To interrogate whether intestinal inflammation is a prerequisite for these effects, we used an inflammation-independent, sporadic CRC tumor model comprising weekly injections of AOM, a potent carcinogen (27), administered for 6 weeks (6xAOM) (Figure 2E). Intriguingly, after 140 days, tumor number and size were equal in *Phd1^–/–^*, *Phd2^+/–^*, *Phd3^–/–^*, and WT control mice (Figure 2F), indicating that in the absence of underlying intestinal inflammation, the loss of any of the PHD isoenzymes does not result in differences concerning tumor number or size of sporadic CRC. Taken together, this demonstrates that intestinal inflammation is required for PHD-dependent tumor growth in CAC.

### The activity of the oncogenic STAT3 and ERK1/2 signaling pathways is increased in colitis-associated Phd2^+/–^ tumors.

**Figure 3:**
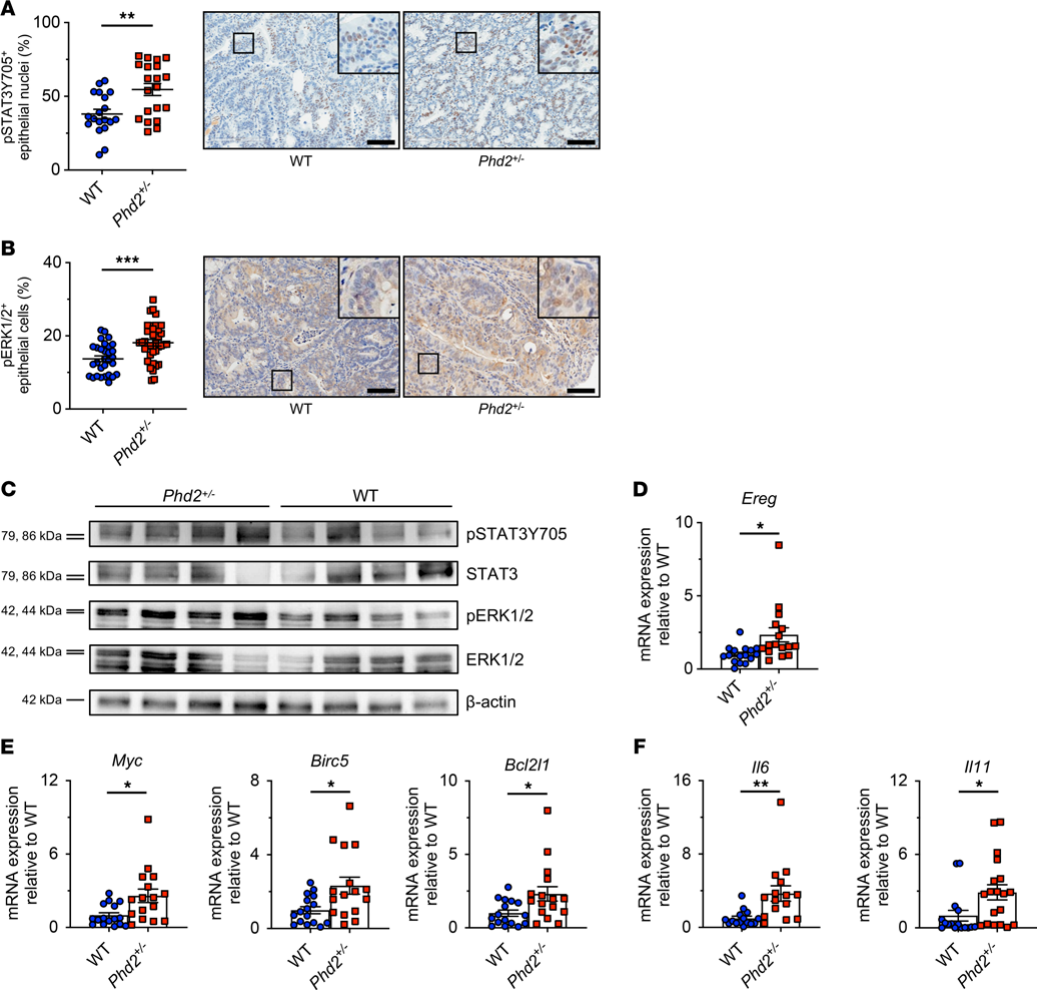
The activity of the oncogenic STAT3 and ERK1/2 signaling pathways is increased in colitis-associated *Phd2^+/–^* tumors. (**A**) Quantification of epithelial nuclear phosphorylated (p-) p-STAT3Y705 immunostaining in WT (*n* = 19) and *Phd2^+/–^* (*n* = 20) tumors and representative histological images (*right*). Scale bar: 100 μm. (**B**) Quantification of epithelial p-ERK1/2 immunostaining in WT (*n* = 30) and *Phd2^+/–^* (*n* = 37) tumors and representative histological images (*right*). Scale bar: 100 μm. (**C**) IB of STAT3, p-STAT3Y705, ERK1/2, and p-ERK1/2 in size- and location-matched WT (*n* = 4) and *Phd2^+/–^* (*n* = 4) tumors. qRT-PCR analysis of EGFR ligand *Ereg* (**D**), STAT3 and ERK1/2 target genes (**E**), and *IL-6* and *IL-11* (**F**) in WT (*n* = 16) and *Phd2^+/–^* (*n* = 16) tumors. Statistical significance was calculated using 1-way ANOVA with Dunnett’s multiple comparisons test in **A** and **B** or 2-tailed Student’s *t* test in **D**–**F**. *P < 0.05, **P < 0.01, ***P < 0.001.

We next sought a possible molecular mechanism promoting tumorigenesis in colitis-associated *Phd2^+/–^* tumors. Since STAT3, ERK1/2, and WNT/β-catenin are key oncogenic signaling pathways in CRC (28–30), we examined phosphorylation of STAT3 and ERK1/2 as well as nuclear localization of β-catenin in the tumor cell compartment of *Phd2^+/–^* and WT control tumors by IHC. Strikingly, *Phd2* haplodeficiency significantly increased STAT3 (Figure 3A) and ERK1/2 phosphorylation (Figure 3B) in epithelial cells of AOM/DSS-induced colon tumors. In contrast, there was no difference in nuclear β-catenin expression in epithelial cells of *Phd2^+/–^* tumors compared with WT controls (Supplemental Figure 3A). The augmented STAT3 and ERK1/2 phosphorylation was also validated by IB (Figure 3C). Of note, there was no difference in STAT3 phosphorylation in epithelial cells of *Phd1^–/–^* tumors compared with WT control tumors as assessed by IHC (Supplemental Figure 3B).

Since the STAT3 and ERK1/2 signaling pathways are downstream of EGFR, we quantified the transcript expression of *Egfr* and all 7 known EGFR ligands — *Ereg*, *Areg*, *Egf*, *Hbegf*, *Tgfa*, *Epgn*, and *Btc* — in colitis-associated tumors from *Phd2^+/–^* and WT control mice by qRT-PCR. Intriguingly, while *Egfr* transcript expression was not significantly altered in *Phd2^+/–^* tumors compared with WT control tumors (Supplemental Figure 3C), *Ereg* was the only EGFR ligand that was significantly upregulated in *Phd2^+/–^* tumors compared with control tumors (Figure 3D and Supplemental Figure 3D). Further validating this, we reanalyzed a publicly available high-density microarray data set that includes transcriptomes from size- and location-matched AOM/DSS-induced and sporadic *Apc^Min/+^* tumors (31). This verified that — in contrast to inflammation-independent *Apc^Min/+^* tumors — *Ereg*, but none of the other EGFR ligands, was significantly upregulated in inflammation-associated tumors compared with the normal control samples (Supplemental Figure 3E). Taken together, this suggests that EREG signaling at least in part contributes to the activation of the oncogenic STAT3 and ERK1/2 signaling pathways in *Phd2^+/–^* tumor cells in CAC.

To further assess activation of the STAT3 and ERK1/2 signaling pathways, we analyzed the mRNA transcript expression of the target genes *Myc*, baculoviral IAP repeat-containing 5 (*Birc5*), and BCL2-like 1 (*Bcl2l1*). Consistent with increased STAT3 and ERK1/2 phosphorylation, transcript expression of these genes was significantly augmented in *Phd2^+/–^* tumors compared with WT control tumors (Figure 3E). Moreover, expression of the proinflammatory cytokines *IL-6* and *IL-11*, which are key protumorigenic mediators in the AOM/DSS model and can signal both via STAT3 and ERK1/2 (28, 32, 33), was increased in *Phd2*^+/–^ tumors compared with WT control tumors (Figure 3F).

Collectively, these results suggest that the enhanced colitis-associated tumorigenesis caused by *Phd2* haplodeficiency is mediated, at least in part, by activation of the STAT3 and ERK1/2 signaling pathways in tumor cells through EREG.

### The number of TAMs in colitis-associated Phd2^+/–^ tumors is increased.

**Figure 4:**
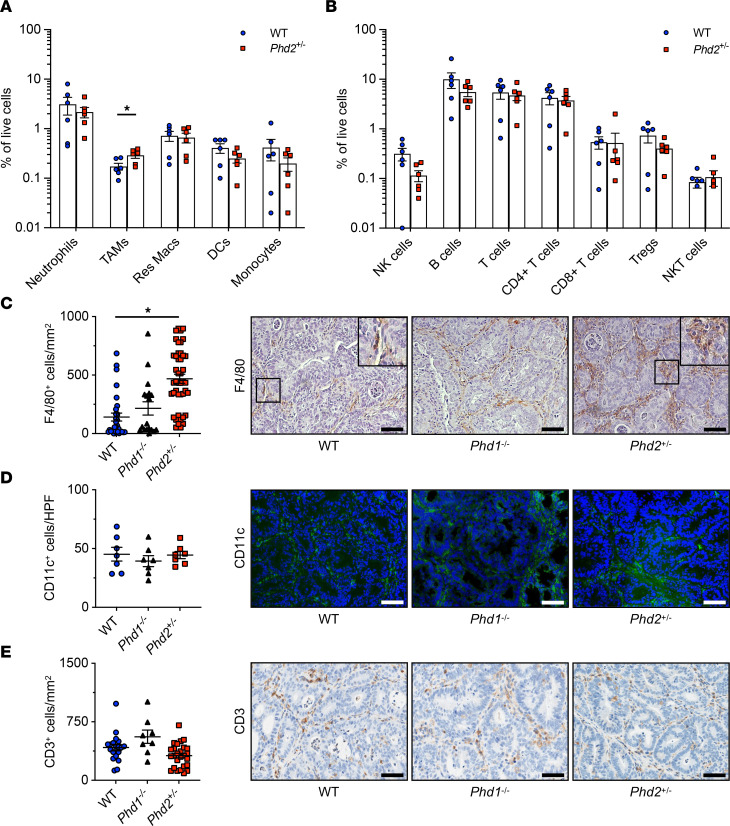
The number of TAMs in colitis-associated *Phd2^+/–^* tumors is increased. Flow cytometry analysis of (**A**) myeloid and (**B**) lymphoid cells in tumors from *Phd2^+/–^* (*n* = 6) and WT (*n* = 6) control mice. (**C**) Quantification of F4/80 immunostaining in WT (*n* = 30), *Phd1^–/–^* (*n* = 18), and *Phd2^+/–^* (*n* = 44) tumors and representative histological images (*right*). Scale bar: 50 μm. (**D**) Quantification of CD11c immunofluorescence staining in WT (*n* = 7), *Phd1^–/–^* (*n* = 7), and *Phd2^+/–^* (*n* = 7) tumors and representative histological images (*right*). Scale bar: 50 μm. (**E**) Quantification of CD3 immunostaining in WT (*n* = 19), *Phd1^–/–^* (*n* = 8), and *Phd2^+/–^* (*n* = 26) tumors and representative histological images (*right*). Scale bar: 50 μm. Statistical significance was calculated using 2-tailed Student’s *t* test in **A** and **B** or 1-way ANOVA with Dunnett’s multiple comparisons test in **C**–**E**. *P < 0.05. HPF, high-power field.

Since immune cells, and specifically myeloid cells such as macrophages, are key determinants of tumor growth in the AOM/DSS model (34), we comprehensively profiled the immune cell landscape of AOM/DSS-induced tumors of *Phd2^+/–^* and WT controls using flow cytometry (Supplemental Figure 4A, and Supplemental Figure 5A). This revealed a significantly increased number of TAMs in AOM/DSS-induced tumors of *Phd2^+/–^* mice compared with WT controls (Figure 4A). Importantly, no other differences in the immune cell composition of *Phd2^+/–^* and WT tumors were detectable (Figure 4, A and B). To further validate these results, we performed IHC and immunofluorescence staining of F4/80-positive macrophages, CD11c-positive DCs, and CD3-positive T cells in AOM/DSS tumors. This supported an increased number of macrophages in tumors of *Phd2^+/–^* mice compared with WT controls (Figure 4C). Consistent with the flow cytometry results, staining of CD11c-positive DCs and CD3-positive T cells showed no differences among the animals (Figure 4, D and E).

To characterize the functional activation state of the TAMs, we assessed the expression of M1 (CD80, CD86, and CCR7) and M2 (CD163 and CD206) polarization markers within the TAM population of *Phd2^+/–^* and WT tumors by flow cytometry. Interestingly, this did not reveal significant changes of macrophage polarization in *Phd2*-deficient macrophages (Supplemental Figure 5B).

Taken together, this indicates that *Phd2* deficiency is associated with an increased number of TAMs in CAC tumors, which could contribute to the increased tumor burden observed in *Phd2^+/–^* mice compared with WT control animals.

### Phd2-deficient BMDMs stimulate tumor proliferation and show increased Ereg expression in vitro.

**Figure 5:**
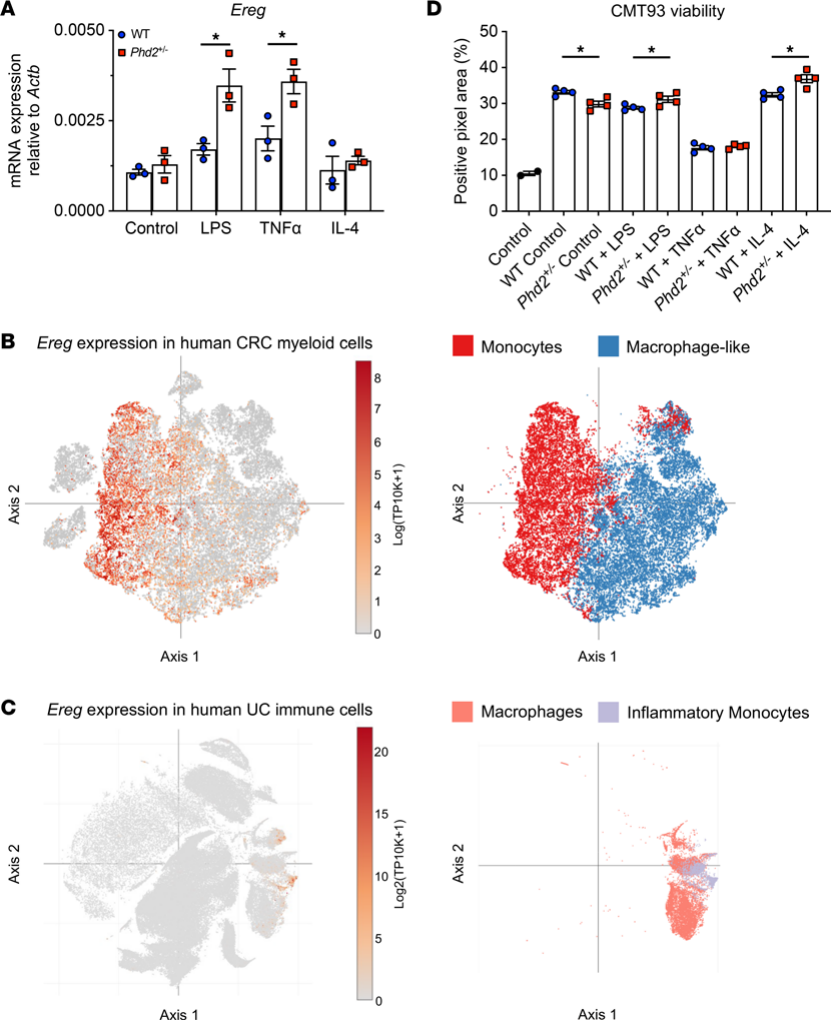
*Phd2*-deficient BMDMs stimulate tumor proliferation and show increased *Ereg* expression in vitro. (**A**) qRT-PCR analysis of *Ereg* in WT and *Phd2^+/–^* BMDMs upon stimulation with control, LPS (100 ng/mL), TNF-α (20 ng/mL), or IL-4 (20 ng/mL) for 24 hours. Dots represent biological replicates. Data represent 4 independent experiments. (**B**) t-Distributed stochastic neighbor embedding (tSNE) plots showing analysis of *Ereg* expression in myeloid cells from human CRC samples. Analysis was performed with publicly available scRNA-Seq data (36). (**C**) tSNE plots showing analysis of *Ereg* expression in immune cells from human UC mucosa samples. Analysis was performed with publicly available scRNA-Seq data (37). (**D**) Crystal violet viability assay of murine CMT-93 rectal cancer cells after 48 hours of treatment with control (RPMI medium + 1% FCS) or conditioned media from BMDMs stimulated with control, LPS, TNF-α, or IL-4. Dots represent technical replicates. Data represent 2 independent experiments. Statistical significance was calculated using Student’s *t* test. *P < 0.05.

After demonstrating that the presence of TAMs is increased and the STAT3 and ERK1/2 pathways are activated in *Phd2^+/–^* tumors in CAC, we next set out to determine how *Phd2* haplodeficiency affects the functionality of macrophages using an established ex vivo model (35). For this, we performed a qRT-PCR analysis of *Phd2^+/–^* and WT control BMDMs, both unstimulated and upon proinflammatory stimulation, with LPS, TNF-α, or IL-4. Strikingly, the transcript expression of *Ereg* was significantly increased 2-fold in *Phd2^+/–^* BMDMs stimulated with LPS or TNF-α compared with WT control BMDMs (Figure 5A), suggesting that TAMs are at least 1 source of the increased *Ereg* expression observed in *Phd2^+/–^* tumors. Consistent with this, interrogation of *Ereg* expression in 2 large-scale single-cell RNA-Seq (scRNA-Seq) data sets of human CRC and UC (Broad Institute) (36, 37) verified that *Ereg* was also expressed in human macrophages and monocytes (Figure 5, B and C), underscoring the importance of EREG in these cells for CAC.

In subsequent studies, we assessed the impact of *Phd2* deficiency of BMDMs on the viability of CRC tumor cells in vitro. For this, we treated murine CMT-93 rectal cancer cells with the supernatant of stimulated *Phd2^+/–^* or WT BMDMs and assessed their viability after 48 hours of treatment. Strikingly, supernatant of *Phd2^+/–^* BMDMs stimulated with LPS or IL-4 significantly increased tumor cell viability of CMT-93 cells compared with treatment with supernatant of WT BMDMs stimulated with LPS or IL-4, indicating that *Phd2^+/–^* macrophages can promote tumor growth in vitro (Figure 5D). Taken together, this suggests that, in addition to their increased presence in AOM/DSS tumors, *Phd2*-deficient macrophages display protumorigenic features and increased Ereg expression in vitro, which implies a mechanistic link to the observed increase in oncogenic STAT3 and ERK1/2 signaling in *Phd2*^+/–^ tumors in vivo.

### Lineage-specific deletion of Phd2 in the hematopoietic but not the epithelial cell compartment aggravates colitis-associated tumor growth.

**Figure 6:**
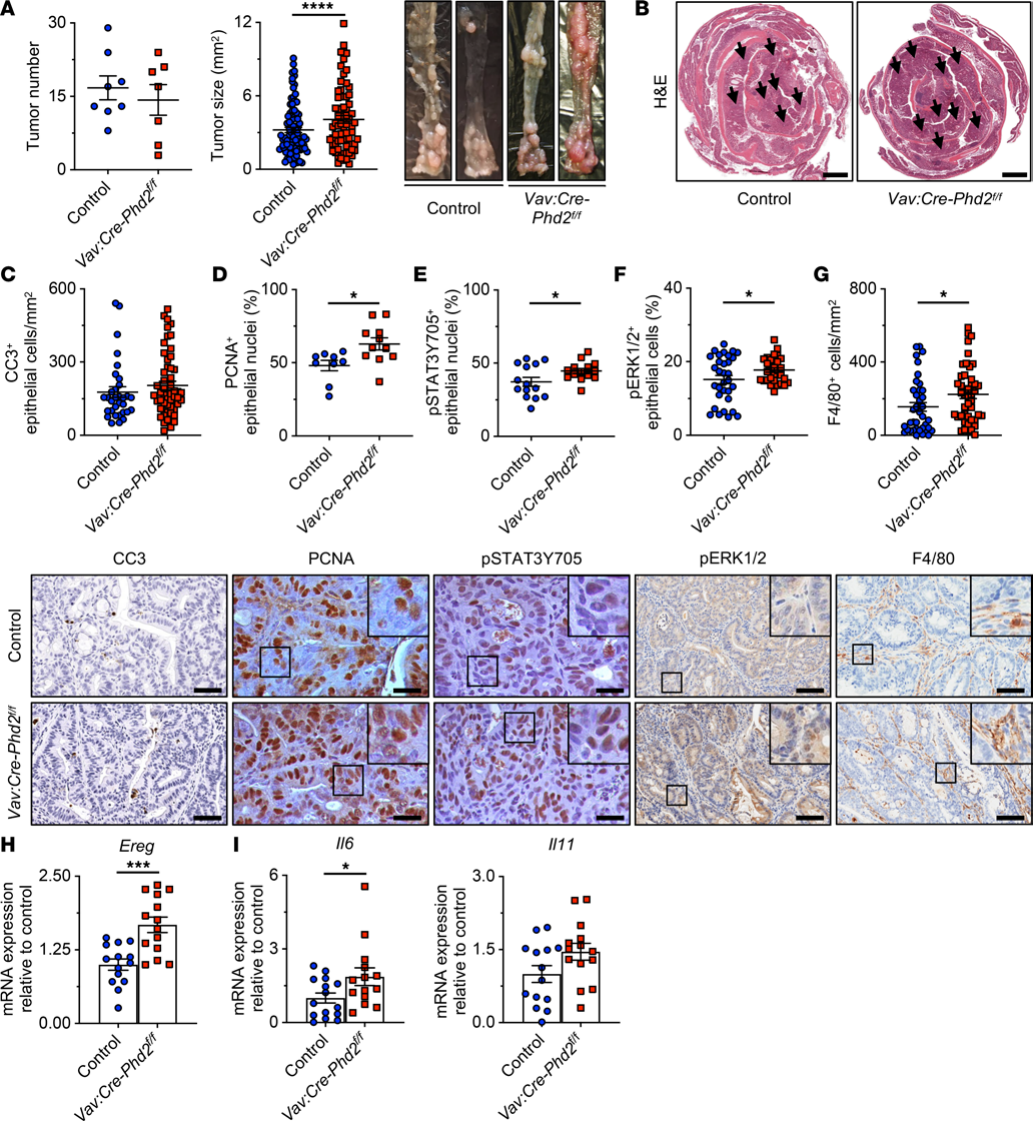
Lineage-specific deletion of *Phd2* in the hematopoietic but not the epithelial cell compartment aggravates colitis-associated tumor growth. (**A**) Macroscopic quantification of AOM/DSS-induced tumors. Number of tumors per mouse (*Phd2^fl/fl^* control: *n* = 8; and *Vav:Cre-Phd2^fl/fl^*: *n* = 7 mice) and size of individual tumors (control: *n* = 127; and *Vav:Cre-Phd2^fl/fl^*: *n* = 100 tumors), and representative macroscopic images (*right*) of colons from control and *Vav:Cre-Phd2^fl/fl^* mice (**B**) H&E staining of colons from control and *Vav:Cre-Phd2^fl/fl^* mice. Arrows indicate colitis-associated tumors. Scale bar: 2 mm. (**C**) Quantification of CC3 immunostaining in control (*n* = 32) and *Vav:Cre-Phd2^fl/fl^* (*n* = 53) tumors (*top*) and representative histological images (*bottom*). Scale bar: 50 μm. (**D**) Quantification of epithelial PCNA immunostaining in control (*n* = 9) and *Vav:Cre-Phd2^fl/fl^* (*n* = 11) tumors (*top*) and representative histological images (*bottom*). Scale bar: 25 μm. (**E**) Quantification of epithelial nuclear p-STAT3Y705 immunostaining in control (*n* = 14) and *Vav:Cre-Phd2^fl/fl^* (*n* = 14) tumors (*top*) and representative histological images (*bottom*). Scale bar: 25 μm. (**F**) Quantification of p-ERK1/2 immunostaining in control (*n* = 31) and *Vav:Cre-Phd2^fl/fl^* (*n* = 30) tumors (*top*) and representative histological images (*bottom*). Scale bar: 100 μm. (**G**) Quantification of F4/80 immunostaining in control (*n* = 38) and *Vav:Cre-Phd2^fl/fl^* (*n* = 45) tumors (*top*) and representative histological images (*bottom*). Scale bar: 100 μm. (**H** and **I**) qRT-PCR analysis of EGFR ligand *Ereg* in **H** and *IL-6* and *IL-11* in **I** in WT (*n* = 14) and *Phd2^+/–^* (*n* = 14) tumors. Statistical significance was calculated using 2-tailed Student’s *t* test. *P < 0.05, ***P < 0.001, ****P < 0.0001.

To test the hypothesis that TAMs are crucial for promoting *Phd2*-deficient tumor growth in CAC, we used transgenic *Vav:Cre-Phd2^fl/fl^* mice that harbor a homozygous deletion of *Phd2* in all hematopoietic lineages (including macrophages) and subjected them to AOM/DSS treatment. Furthermore, to exclude a potential impact of PHD2 in epithelial cells on CAC growth, we also induced AOM/DSS tumors in *Villin:Cre-Phd2^fl/fl^* mice, which are *Phd2* deficient in the intestinal epithelial cells (IECs) of the small intestine and colon (38, 39). In line with our previous results with *Phd2^+/–^* mice, *Vav:Cre-Phd2^fl/fl^* mice displayed significantly bigger tumors, while the tumor number was not changed compared with control animals (Figure 6, A and B). While tumors of *Vav:Cre-Phd2^fl/fl^* and control mice were equally apoptotic as assessed by CC3 IHC staining (Figure 6C), tumors from *Vav:Cre-Phd2^fl/fl^* mice showed aggravated tumor proliferation compared with their controls as assessed by IHC for PCNA (Figure 6D). Moreover, IHC staining revealed significantly increased phosphorylation of STAT3 and ERK1/2 (Figure 6, E and F) but unchanged nuclear β-catenin expression in *Vav:Cre-Phd2^fl/fl^* tumors compared with the control tumors (Supplemental Figure 6A). Strikingly, consistent with the phenotype observed in *Phd2^+/–^* animals, IHC staining for F4/80 suggested a significant increase in the number of TAMs in *Vav:Cre-Phd2^fl/fl^* tumors compared with the control tumors (Figure 6G). Moreover, upstream of STAT3 and ERK1/2 signaling, the mRNA transcript level of EGFR ligand *Ereg*, as quantified by qRT-PCR, was significantly augmented (Figure 6H), while the expression of *Egfr* and all other EGFR ligands — *Areg*, *Egf*, *Hbegf*, *Tgfa*, *Epgn*, and *Btc* — was not significantly altered in *Vav:Cre-Phd2^fl/fl^* tumors compared with control tumors (Supplemental Figure 6, B and C). Furthermore, the transcript expression of protumorigenic *IL-6* was significantly increased, while expression of *IL-11* was modestly (but not significantly) elevated in *Vav:Cre-Phd2^fl/fl^* tumors compared with WT control tumors (Figure 6I).

In contrast, *Villin:Cre-Phd2^fl/fl^* mice did not display significantly altered CAC tumor growth compared with control animals (Supplemental Figure 7, A and B). Consistently, tumor proliferation, apoptosis, and STAT3 phosphorylation (Supplemental Figure 7, C–E), as well as gene expression of *Ereg* (Supplemental Figure 7F), *IL-6*, and *IL-11* (Supplemental Figure 7G), were not significantly altered in *Villin:Cre-Phd2^fl/fl^* mice as compared with the controls. Together, *Phd2* deficiency in hematopoietic cells (including TAMs), but importantly not in IECs, promotes CAC tumor growth at least in part by activation of the STAT3 and ERK1/2 signaling pathways mediated by EREG.

**Figure 7:**
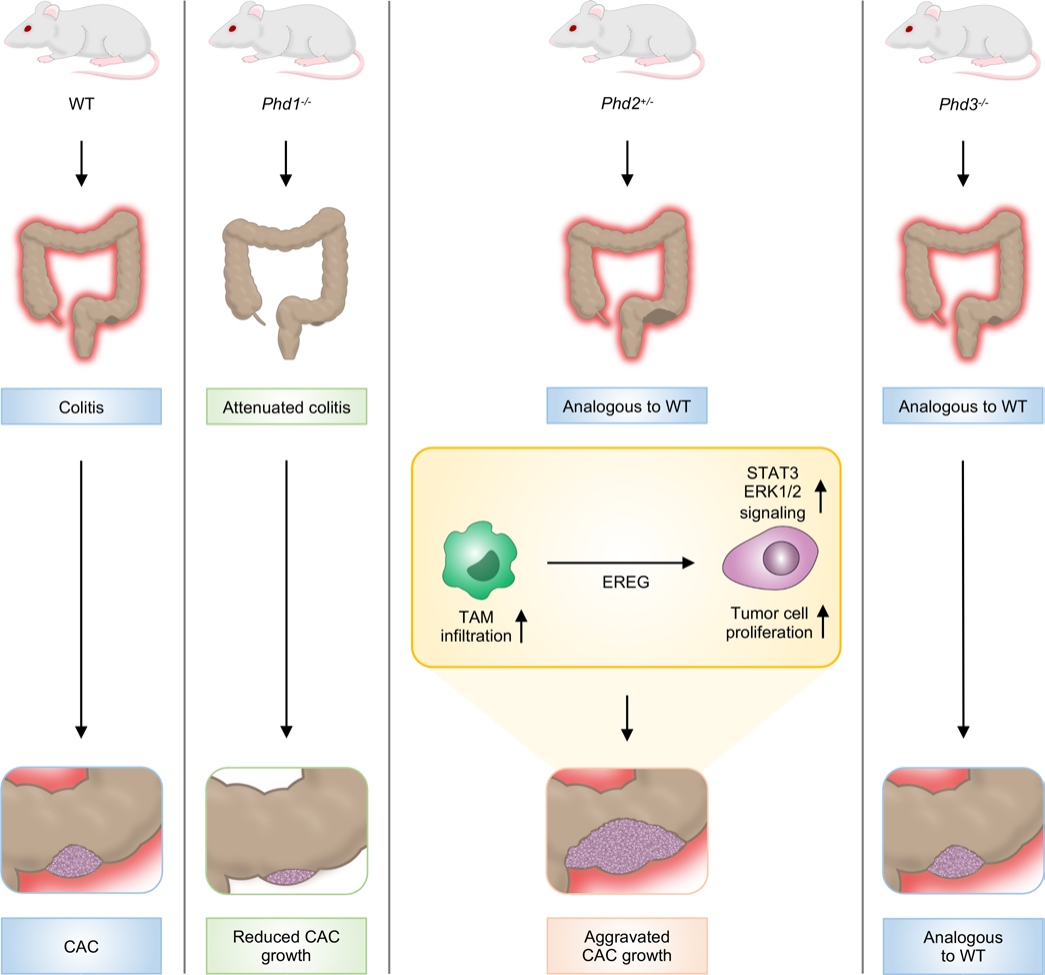
The HIF-prolyl hydroxylases have distinct and nonredundant roles in colitis-associated cancer. PHD1–3 have distinct effects on CAC growth, which is tumor promoting (PHD1), tumor inhibiting (PHD2), or neutral (PHD3). Intestinal inflammation is diminished in *Phd1*-deficient but unaltered in *Phd2-* and *Phd3*-deficient mice. In CAC, PHD2 deficiency (i) increases the number of TAMs, (ii) promotes *Ereg* expression in macrophages, and (iii) augments STAT3 and ERK1/2 signaling, which at least in part contributes to aggravated tumor cell proliferation in colitis-associated tumors.

In conclusion, each of the 3 different HIF-PHD isoenzymes PHD1–3 has a distinct impact on CAC but importantly not on inflammation-independent, sporadic CRC tumor growth. This effect is tumor promoting (PHD1), tumor inhibiting (PHD2), or neutral (PHD3) (Figure 7). PHD2 expression (i) reduces the number of TAMs in AOM/DSS tumors, (ii) impairs the protumorigenic properties of macrophages at least in part through decreased *Ereg* expression, and (iii) diminishes STAT3 and ERK1/2 signaling in colitis-associated tumors.

## Discussion

Despite the significant advances in treatment for IBD, current treatment options are still limited. The occurrence of CAC is positively correlated with the severity, extent, and duration of intestinal inflammation (3, 4), and, thus, there is an ongoing and unmet need for innovative therapeutic strategies. The HIF-PHD1–3 isoenzymes are increasingly considered as therapeutic targets (40, 41); however, a comprehensive analysis of their biological role in the pathogenesis of CAC has been lacking. Here, we demonstrate that PHD1–3 affect the 2 key features of CAC, chronic colitis and colorectal tumorigenesis, in a nonredundant and context-dependent manner, which conceptually advances current mechanistic understanding of PHD enzyme functions in these 2 important areas.

In a model of AOM/DSS-triggered chronic colitis, loss of *Phd1* (but not *Phd2* or *Phd3*) mitigated colonic inflammation, resulting in reduced disease activity. This extends recently published data from our group and others on the protective effect of *Phd1* deficiency in chronic colitis (15, 16). Strikingly, *Phd1^–/–^* mice treated with AOM/DSS also displayed significantly reduced tumor formation. This phenotype was not caused by alterations in apoptosis, proliferation, or immune cell infiltration in tumors, suggesting that *Phd1* deficiency affects tumor initiation or early tumor propagation rather than progression of established tumors. Importantly, when animals were treated with AOM alone, there was no effect on intestinal tumor burden, indicating DSS-induced intestinal inflammation is required to induce the phenotypical differences observed between *Phd1^–/–^* and control mice. Mechanistically, in DSS-induced colitis, *Phd1* deficiency (i) diminishes apoptosis of enterocytes upon DSS treatment and, thus, increases the intestinal epithelial barrier function (EBF) (15) and (ii) skews macrophage polarization toward an M2 antiinflammatory phenotype (16). Here, we hypothesize that in our AOM/DSS-induced model of CAC, *Phd1* deficiency stabilizes the intestinal EBF and, thus, impairs the establishment of a proinflammatory mucosal milieu, which is required to promote tumorigenesis in this model (42). Consistently, mucosal concentrations of proinflammatory cytokines, such as IL-1β, IL-6, and TNF-α, have been shown to be significantly reduced in *Phd1^–/–^* animals upon DSS treatment (15). This highlights the profound antiinflammatory effects of *Phd1* deficiency in colorectal mucosa, which most likely leads to diminished tumor growth observed in these mice.

*Phd3* deficiency, by contrast, did not have an impact on colitis activity or on the formation of colitis-associated tumors. The latter is in apparent contrast to previously published data, which revealed a tumor-suppressive role of PHD3 in CRC (21, 22). However, these previous studies focused solely on tumor cell–autonomous effects of PHD3 in heterotopic or orthotopic tumor models (21, 22), while in our present study we subjected global *Phd3*-KO mice to colitis-associated and sporadic colorectal tumor models, thus assessing the biological relevance of PHD3 in both tumor cells and the tumor microenvironment (TME). In that respect, our data suggest that loss of *Phd3* in the TME exerts antitumorigenic effects that may compensate for putative protumorigenic effects of *Phd3* deficiency in tumor cells. In keeping with this, we have previously demonstrated that *Phd3*-deficient macrophages display an increased expression of M1 polarization markers (43). Intriguingly, M1-polarized macrophages have been proposed to be critical for tumor growth suppression in the AOM/DSS-induced CAC model (44, 45).

In contrast to *Phd1* or *Phd3* deficiency, *Phd2* haplodeficiency promoted CAC tumor growth in the AOM/DSS model. Similar to the phenotype observed in *Phd1*-deficient mice, protumorigenic effects of *Phd2* haplodeficiency mediated by AOM/DSS treatment were abrogated when animals were treated with AOM alone, indicating that DSS-induced inflammation is a crucial requirement for this tumor-promoting effect. This tumor-promoting role of *Phd2* deficiency in inflammation-associated cancer is in line with recent studies in inflammation-associated hepatocarcinogenesis (46).

Mechanistic analyses revealed that oncogenic STAT3 and ERK1/2 signaling, which are critical for promotion of CAC (28, 31), are enhanced in *Phd2^+/–^* tumors compared with their WT counterparts. Consistently, the expression of several downstream target genes of STAT3 and ERK1/2 were increased in *Phd2^+/–^* tumors. Moreover, the expression of the EGFR ligand EREG as well as proinflammatory cytokines IL-6 and IL-11, which can all induce phosphorylation of STAT3 and ERK1/2 and create a protumorigenic environment in the AOM/DSS model (28, 31–33, 47), are augmented in *Phd2^+/–^* tumors. Because IL-6 and IL-11 are also target genes of active STAT3 and ERK1/2 signaling (32, 48) and Ereg expression can be induced by IL-6 (49), this suggests an intimate crosstalk between IL-6, IL-11, and EREG signaling pathways that, in our model, contributed to increased colitis-associated tumor growth in *Phd2*-deficient animals. Intriguingly, EREG has been shown to be secreted by myeloid cells in the TME (50) and is a predominant regulatory factor for the growth of CAC (31). Therefore, we performed a comprehensive analysis of the immune cell landscape of AOM/DSS-induced tumors, which revealed a markedly increased presence of TAMs in *Phd2^+/–^* tumors compared with WT controls. This suggested that the enhanced number of TAMs contributes to increased tumor growth in these tumors. Loss of *Phd2* has been recently shown to boost the migratory capacity of another subset of myeloid cells, neutrophils, and promote neutrophil invasion into inflamed tissues (51). Hence, it is conceivable that *Phd2* deficiency also positively affects invasion of TAMs into AOM/DSS tumors. In line with previous studies that indicate that TAMs display a great diversity of functional activation states beyond the M1/M2 dichotomy and macrophages with different phenotypes coexist within the same TAM population (52, 53), *Phd2*-deficient TAMs did not show a clear polarization.

Beyond the increased number of TAMs in *Phd2^+/–^* tumors, our in vitro studies also demonstrated that stimulated *Phd2^+/–^* BMDMs had increased transcript levels of *Ereg*, suggesting *Phd2*-deficient TAMs are one of the cellular sources for increased levels of protumorigenic *Ereg* in *Phd2^+/–^* tumors. Also, our in vitro studies show that the supernatant of *Phd2*-deficient BMDMs significantly increases the viability of a CRC cell line compared with WT BMDMs, providing additional evidence that *Phd2* deficiency in TAMs contributes to augmented growth of *Phd2^+/–^* colitis-associated tumors.

To further interrogate the importance of TAMs for *Phd2*-deficient tumor growth in CAC, we employed mice deficient in *Phd2* in all hematopoietic cells or IECs. Strikingly, *Vav:Cre-Phd2^fl/fl^* mice displayed a tumor-promoting phenotype upon AOM/DSS treatment with increased tumoral activity of STAT3 and ERK1/2, confirming the notion that these 2 pathways at least contribute to PHD2-mediated CAC tumor development. Moreover, TAM presence was substantially increased, which indicates that loss of *Phd2* in hematopoietic cells including TAMs is required for the tumor-promoting phenotype in *Phd2^+/–^* animals. In contrast, *Villin:Cre-Phd2^fl/fl^* mice did not display any alterations in tumor growth, proliferation, apoptosis, or STAT3 activation, suggesting that IEC-specific *Phd2* expression is dispensable for AOM/DSS-induced tumor growth, which validates work by others (54). In concert, these results demonstrate that macrophages critically contribute to the tumor-promoting phenotype observed in *Phd2^+/–^* and *Vav:Cre-Phd2^fl/fl^* animals.

Taken together, we propose that *Phd2* deficiency promotes CAC tumor growth in at least 2 ways: an increase in the number and protumorigenic function of TAMs associated with an augmented expression of *Ereg*, which contributes to oncogenic STAT3 and ERK1/2 signaling and, thus, aggravates tumor proliferation. Although our data indicate that TAMs and EREG play a crucial role in controlling tumor growth in CAC, we cannot rule out that *Phd2* deficiency might also influence tumor development through additional cell types in the TME, such as fibroblasts (31), and additional growth factors. Conditional KO models that disrupt PHD in various cells of the TME could provide additional insights into how PHD2 affects the carcinogenesis of CAC.

To conclude, we have demonstrated that PHD1–3 have distinct and nonredundant biological roles in the pathogenesis of CAC. Moreover, we have revealed a tumor-inhibiting role of PHD2 in CAC. These findings are particularly important considering the promising advent of PHD inhibitors in the clinical setting (40, 41). Small-molecule pan-PHD inhibitors have already been approved for patients with renal anemia and are currently being evaluated in clinical phase II trials for UC (55–58). Given the pleiotropic and complex nature of the PHD/HIF signaling pathway, it seems desirable that clinically used PHD inhibitors are isoenzyme selective, especially considering their use in chronic diseases such as IBD. Nevertheless, to date there are no small-molecule PHD inhibitors available specifically targeting single isoenzymes. Therefore, a mechanistic understanding of the divergent biological functions of PHD1–3 is critical for the clinical administration of pan-PHD inhibitors and the development of isotype-specific small-molecule inhibitors.

## Methods

Supplemental Methods are available online with this article.

### Mice.

Homozygous *Phd1^–/–^* and *Phd3^–/–^* as well as heterozygous *Phd2^+/–^* mice of a mixed 129/Sv/Swiss background have been described previously (15). *Phd2^+/–^* mice were used because homozygous *Phd2^–/–^* mice are not viable (24). Control animals were a mix of WT littermates from *Phd1^–/–^*, *Phd2^+/–^*, and *Phd3^–/–^* lines, which were confirmed to display similar clinical and molecular alterations (as determined by qRT-PCR of several proinflammatory cytokines) upon DSS challenge.

The *Villin:Cre* transgenic mouse line was obtained from the Jackson Laboratories and crossed with floxed *Phd2^fl/fl^* mice at the Dresden University of Technology to create *Villin:Cre-Phd2^fl/fl^* mice (59). The *Vav:Cre-Phd2^fl/fl^* mice have been described previously (60). Both mouse strains were maintained on a C57BL/6 background. Littermate *Phd2^fl/fl^* mice negative for Cre recombinase were used as controls.

In all studies, 8- to 12-week-old female and male mice with a mean weight of 32 g were equally distributed among experimental groups. Up to 5 animals were group housed in standard laboratory cages under specific pathogen–free conditions in a temperature-controlled room at 22°C with free access to water and commercial chow ad libitum with a 12-hour light/dark cycle.

### Mouse models of CAC and sporadic CRC.

To induce CAC, mice were i.p. injected with 10 mg/kg BW AOM (Sigma-Aldrich, catalog A5486) and subsequently treated with 3 cycles of DSS (36,000–50,000 MW; MP Biomedicals, catalog 0216011010) starting on day 7. *Phd1^–/–^*, *Phd2^+/–^*, *Phd3^–/–^*, and respective control animals received 2.5% DSS in the drinking water for 7 days (first 2 cycles) or 5 days (third cycle) ad libitum followed by a recovery phase of 14 or 16 days, respectively. Animals were sacrificed 4 weeks after initiation of the last DSS cycle.

*Villin:Cre-Phd2^fl/fl^*, *Vav:Cre-Phd2^fl/fl^*, and respective control animals were treated with 3 cycles of 1.5% DSS for 5 days and sacrificed 16 days after the last cycle. The DAI, comprising relative BW loss, stool consistency, and rectal bleeding, was recorded as previously described (15).

For studies on inflammation-independent, sporadic colorectal carcinogenesis, *Phd1^–/–^*, *Phd2^+/–^*, *Phd3^–/–^*, and control animals were subjected to weekly injections of 10 mg/kg BW AOM for 6 weeks (6xAOM). Experiments were terminated 20 weeks after study commencement.

At the end of all experiments or when animals reached endpoint criteria (BW loss > 20% or clinical signs of persistent distress or pain), animals were euthanized by cervical dislocation. The colon was removed and flushed with PBS. The length between the ileocecal junction and the proximal rectum was measured. Pictures of the colon were taken with a Leica M651 Surgical Microscope (Leica Microsystems), and tumor number and size were analyzed using ImageJ (NIH) for MacOS (Version 1.52q).

### Flow cytometry.

AOM/DSS tumors were minced using scalpels and surgical scissors and then incubated in 15 mL Falcon tubes in a total of 5 mL RPMI-1640 Medium (Sigma-Aldrich, catalog R8758) containing 2% FCS, 3 mg/mL Collagenase D (Roche, catalog 11088858001) and 0.5 mg/mL DNase I (Roche, catalog 11284932001) in an orbital shaker incubator at 130 rpm and 37°C for 75 minutes in a horizontal position. Digested tumors were passed twice through a 70 μm cell strainer (Corning) to obtain single-cell suspensions. Viability was assessed by trypan blue staining, and single-cell suspensions were adjusted to a concentration of 1 × 10^7^ live cells/mL. Single-cell suspensions were incubated with mouse FcR Blocking Reagent (Miltenyi Biotec, catalog 130-092-575) and then stained with 2 antibody panels to identify lymphoid and myeloid cells, respectively (for antibodies, see Supplemental Methods). Flow cytometry data were acquired on a BD LSRFortessa and analyzed using FlowJo (Version 10.8.1, BD). We employed a previously published gating strategy to identify myeloid populations in AOM/DSS tumors (61). Splenic and mucosal single-cell preparations were used to validate gate settings.

### Tumor cell viability assay.

Conditioned media (CM) from WT and *Phd2^+/–^* BMDMs stimulated with 100 ng/mL LPS, 20 ng/mL TNF-α, or 20 ng/mL IL-4 for 24 hours were sterile filtered, frozen, and stored at –20°C. Murine CMT-93 rectal cancer cells (ATCC, catalog CCL-223) were grown in DMEM supplemented with 10% FCS and 1% penicillin/streptomycin. Prior to experiments, CMT-93 cells were seeded at a density of 10,000 cells per well into 48-well plates and allowed to settle down and adhere for 6 hours. Then, CMT-93 cells were treated with control (RPMI medium + 1% FCS) or CM from BMDMs stimulated with control, LPS-, TNF-α– or IL-4–containing medium. After 48 hours of treatment, cells were washed with PBS once and incubated with crystal violet (MilliporeSigma) at room temperature for 15 minutes to stain viable cells. After 3 rounds of PBS washing and drying, microscopic photos were taken of each well, and the stained area per well was quantified using ImageJ. Experiments were performed in 2–4 technical replicates and repeated twice.

### Statistics.

All in vivo data represent 2–4 independently performed experiments. Continuous data sets from 2 groups or more were analyzed by Student’s *t* test or ANOVA with appropriate post hoc test, respectively. Student’s *t* tests were 2 tailed, and 1-way ANOVA was performed if not otherwise indicated in the figure legends. All data sets were tested for normal distribution. If data were not normally distributed, appropriate nonparametric tests were performed as indicated in the figure legends. All statistical analyses were performed using GraphPad Prism for MacOS (Version 8.0.2; GraphPad Software). Data are presented as the mean ± SEM. A *P* value less than 0.05 was considered significant. *P* values and statistical tests used are indicated in the figure legends.

### Study approval.

Animal experiments were approved by the local animal welfare committees (Regierungspräsidium Karlsruhe, Karlsruhe, Germany [G263-14]; and Landesdirektion Sachsen, Dresden, Germany [TVV09/2014]), performed in accordance with the NIH *Guide for the Care and Use of Laboratory Animals* (NIH Publication No. 8023, revised 1978) (see also ARRIVE checklist) and carried out at the Interfaculty Biomedical Facility of the University of Heidelberg and at the Dresden University of Technology.

## Author contributions

KBK, JB, CTT, M Schneider, and JMH designed the study. KBK, JB, PR, M Salfenmoser, JMG, and BW conducted the animal experiments. KBK, JB, PR, NAG, TG, M Salfenmoser, JMG, MJS, and BW performed further data acquisition and analysis. KBK, JB, M Schneider, and JMH designed the experiments, interpreted the data, and cowrote the manuscript with input from all authors. KBK and JB are co–first authors and contributed equally to the manuscript. The authorship order of the co–first authors was assigned by flipping a coin. All authors approved of the final manuscript version and the authorship order.

## Supplementary Material

Supplemental data

## Version 1 11/22/2022

Electronic publication

## References

[1] Jess T (2006). Risk of intestinal cancer in inflammatory bowel disease: a population-based study from Olmsted county, Minnesota. Gastroenterology.

[2] Ekbom A (1990). Ulcerative colitis and colorectal cancer. A population-based study. N Engl J Med.

[3] Gupta RB (2007). Histologic inflammation is a risk factor for progression to colorectal neoplasia in ulcerative colitis: a cohort study. Gastroenterology.

[4] Rutter M (2004). Severity of inflammation is a risk factor for colorectal neoplasia in ulcerative colitis. Gastroenterology.

[5] Derikx L (2016). Risk of neoplasia after colectomy in patients with inflammatory bowel disease: a systematic review and meta-analysis. Clin Gastroenterol Hepatol.

[6] Leowardi C (2016). Prognosis of ulcerative colitis-associated colorectal carcinoma compared to sporadic colorectal carcinoma: a matched pair analysis. Ann Surg Oncol.

[7] Lamb CA (2019). British Society of Gastroenterology consensus guidelines on the management of inflammatory bowel disease in adults. Gut.

[8] Ordas I (2012). Ulcerative colitis. Lancet.

[9] Cummins EP (2013). Hydroxylases as therapeutic targets in inflammatory bowel disease. Lab Invest.

[10] Schofield CJ, Ratcliffe PJ (2004). Oxygen sensing by HIF hydroxylases. Nat Rev Mol Cell Biol.

[11] Semenza GL (2011). Oxygen sensing, homeostasis, and disease. N Engl J Med.

[12] Karhausen J (2005). Inflammatory hypoxia: role of hypoxia-inducible factor. Cell Cycle.

[13] Karhausen J (2004). Epithelial hypoxia-inducible factor-1 is protective in murine experimental colitis. J Clin Invest.

[14] Van Welden S (2013). Differential expression of prolyl hydroxylase 1 in patients with ulcerative colitis versus patients with Crohn’s disease/infectious colitis and healthy controls. J Inflamm (Lond).

[15] Tambuwala MM (2010). Loss of prolyl hydroxylase-1 protects against colitis through reduced epithelial cell apoptosis and increased barrier function. Gastroenterology.

[16] Van Welden S (2017). Haematopoietic prolyl hydroxylase-1 deficiency promotes M2 macrophage polarization and is both necessary and sufficient to protect against experimental colitis. J Pathol.

[17] Harnoss JM (2020). Prolyl hydroxylase inhibition mitigates pouchitis. Inflamm Bowel Dis.

[18] Cummins EP (2008). The hydroxylase inhibitor dimethyloxalylglycine is protective in a murine model of colitis. Gastroenterology.

[19] Robinson A (2008). Mucosal protection by hypoxia-inducible factor prolyl hydroxylase inhibition. Gastroenterology.

[20] Erez N (2003). Expression of prolyl-hydroxylase-1 (PHD1/EGLN2) suppresses hypoxia inducible factor-1alpha activation and inhibits tumor growth. Cancer Res.

[21] Radhakrishnan P (2016). Prolyl hydroxylase 3 attenuates MCL-1-mediated ATP production to suppress the metastatic potential of colorectal cancer cells. Cancer Res.

[22] Xue J (2010). Prolyl hydroxylase-3 is down-regulated in colorectal cancer cells and inhibits IKKbeta independent of hydroxylase activity. Gastroenterology.

[23] Chan DA (2009). Tumor vasculature is regulated by PHD2-mediated angiogenesis and bone marrow-derived cell recruitment. Cancer Cell.

[24] Mazzone M (2009). Heterozygous deficiency of PHD2 restores tumor oxygenation and inhibits metastasis via endothelial normalization. Cell.

[25] Berra E (2003). HIF prolyl-hydroxylase 2 is the key oxygen sensor setting low steady-state levels of HIF-1alpha in normoxia. EMBO J.

[26] Wirtz S (2017). Chemically induced mouse models of acute and chronic intestinal inflammation. Nat Protoc.

[27] Takahashi M, Wakabayashi K (2004). Gene mutations and altered gene expression in azoxymethane-induced colon carcinogenesis in rodents. Cancer Sci.

[28] Grivennikov S (2009). IL-6 and Stat3 are required for survival of intestinal epithelial cells and development of colitis-associated cancer. Cancer Cell.

[29] Cancer Genome Atlas Network (2012). Comprehensive molecular characterization of human colon and rectal cancer. Nature.

[30] Fang JY, Richardson BC (2005). The MAPK signalling pathways and colorectal cancer. Lancet Oncol.

[31] Neufert C (2013). Tumor fibroblast-derived epiregulin promotes growth of colitis-associated neoplasms through ERK. J Clin Invest.

[32] Jarnicki A (2010). Stat3: linking inflammation to epithelial cancer - more than a “gut” feeling?. Cell Div.

[33] Putoczki TL (2013). Interleukin-11 is the dominant IL-6 family cytokine during gastrointestinal tumorigenesis and can be targeted therapeutically. Cancer Cell.

[34] Bader JE (2018). Macrophage depletion using clodronate liposomes decreases tumorigenesis and alters gut microbiota in the AOM/DSS mouse model of colon cancer. Am J Physiol Gastrointest Liver Physiol.

[35] Trouplin V (2013). Bone marrow-derived macrophage production. J Vis Exp.

[36] Pelka K (2021). Spatially organized multicellular immune hubs in human colorectal cancer. Cell.

[37] Smillie CS (2019). Intra- and inter-cellular rewiring of the human colon during ulcerative colitis. Cell.

[38] Georgiades P (2002). VavCre transgenic mice: a tool for mutagenesis in hematopoietic and endothelial lineages. Genesis.

[39] El Marjou F (2004). Tissue-specific and inducible Cre-mediated recombination in the gut epithelium. Genesis.

[40] Harnoss JM (2015). Therapeutic inhibition of prolyl hydroxylase domain-containing enzymes in surgery: putative applications and challenges. Hypoxia (Auckl).

[41] Bhat S, Rieder F Hypoxia-inducible factor 1-alpha stabilizers in the treatment of inflammatory bowel diseases: oxygen as a novel IBD therapy. J Crohns Colitis.

[42] De Robertis M (2011). The AOM/DSS murine model for the study of colon carcinogenesis: from pathways to diagnosis and therapy studies. J Carcinog.

[43] Kiss J (2012). Loss of the oxygen sensor PHD3 enhances the innate immune response to abdominal sepsis. J Immunol.

[44] Singh K (2018). Ornithine decarboxylase in macrophages exacerbates colitis and promotes colitis-associated colon carcinogenesis by impairing M1 immune responses. Cancer Res.

[45] Eom YW (2020). M1 macrophages promote TRAIL expression in adipose tissue-derived stem cells, which suppresses colitis-associated colon cancer by increasing apoptosis of CD133^+^ cancer stem cells and decreasing M2 macrophage population. Int J Mol Sci.

[46] Heindryckx F (2012). Effect of prolyl hydroxylase domain-2 haplodeficiency on the hepatocarcinogenesis in mice. J Hepatol.

[47] Wang Y (2019). Epiregulin reprograms cancer-associated fibroblasts and facilitates oral squamous cell carcinoma invasion via JAK2-STAT3 pathway. J Exp Clin Cancer Res.

[48] Leonard M (1999). Role of MAP kinase pathways in mediating IL-6 production in human primary mesangial and proximal tubular cells. Kidney Int.

[49] Kyotani Y (2018). Intermittent hypoxia-induced epiregulin expression by IL-6 production in human coronary artery smooth muscle cells. FEBS Open Bio.

[50] Vlaicu P (2013). Monocytes/macrophages support mammary tumor invasivity by co-secreting lineage-specific EGFR ligands and a STAT3 activator. BMC Cancer.

[51] Sormendi S (2021). HIF2α is a direct regulator of neutrophil motility. Blood.

[52] Guentsch A (2017). PHD2 is a regulator for glycolytic reprogramming in macrophages. Mol Cell Biol.

[53] Locati M (2020). Diversity, mechanisms, and significance of macrophage plasticity. Annu Rev Pathol.

[54] Xie Y (2018). Prolyl hydroxylase 2 is dispensable for homeostasis of intestinal epithelium in mice. Acta Biochim Biophys Sin (Shanghai).

[55] Chen N (2019). Roxadustat treatment for anemia in patients undergoing long-term dialysis. N Engl J Med.

[56] Chen N (2019). Roxadustat for anemia in patients with kidney disease not receiving dialysis. N Engl J Med.

[57] Van Welden S (2017). Intestinal hypoxia and hypoxia-induced signalling as therapeutic targets for IBD. Nat Rev Gastroenterol Hepatol.

[58] Danese S (2022). Randomised clinical trial: a phase 1b study of GB004, an oral HIF-1α stabiliser, for treatment of ulcerative colitis. Aliment Pharmacol Ther.

[59] Singh RP (2013). HIF prolyl hydroxylase 2 (PHD2) is a critical regulator of hematopoietic stem cell maintenance during steady-state and stress. Blood.

[60] Rauner M (2016). Increased EPO levels are associated with bone loss in mice lacking PHD2 in EPO-producing cells. J Bone Miner Res.

[61] Olesch C (2020). S1PR4 ablation reduces tumor growth and improves chemotherapy via CD8+ T cell expansion. J Clin Invest.

[62] Katakura K (2005). Toll-like receptor 9-induced type I IFN protects mice from experimental colitis. J Clin Invest.

