# Supplementary material for: The HIF-prolyl hydroxylases have distinct and nonredundant roles in colitis-associated cancer
Source: JCI Insight. 2022 Nov 22;7(22):e153337. doi: 10.1172/jci.insight.153337 (PMC9746807; doi:10.1172/jci.insight.153337)
Supplement: Supplemental data [file jciinsight-7-153337-s054.pdf]

Supplemental Material for

The HIF-prolyl hydroxylases have distinct and non-redundant roles in colitis-associated cancer

Kilian B. Kennel[1], Julius Burmeister[1], Praveen Radhakrishnan[1], Nathalia A. Giese[1], Thomas Giese[2], Martin Salfenmoser[1], Jasper M. Gebhardt[1], Moritz J. Strowitzki[1], Cormac T. Taylor[3], Ben Wielockx[4], Martin Schneider[1], and Jonathan M. Harnoss[1,*]

[1]Department of General, Visceral and Transplantation Surgery, University Hospital Heidelberg, Heidelberg, Germany
[2]Institute of Immunology, University Hospital Heidelberg, Heidelberg, Germany
[3]School of Medicine, Systems Biology Ireland and the Conway Institute of Biomolecular and Biomedical Research, University College Dublin, Dublin, Ireland
[4]Institute for Clinical Chemistry and Laboratory Medicine, Dresden University of Technology, Dresden, Germany

[*]Corresponding author: Department of General, Visceral and Transplantation Surgery, Heidelberg University, Im Neuenheimer Feld 420, 69120 Heidelberg. Phone: +49-6221-566110; Email: Jonathan.harnoss@med.uni-heidelberg.de

Authorship note: KBK and JB are co-first authors and contributed equally to the manuscript. MSch and JMH are co-last authors and contributed equally to the manuscript.

This file includes:

- Supplemental Methods
- Supplemental Figures 1-7 including Figure legends
- Supplemental Tables 1 and 2
- References for Supplemental Data

SUPPLEMENTAL METHODS

Histology, immunohistochemistry, immunofluorescence

For histology and IHC, tumor-bearing colons were fixed as “swiss rolls” using formalin and ethanol, embedded in paraffin, and cut at 5 µm thickness (1). A subset of tumors was frozen and processed for cryosectioning and subsequent IF staining. Paraffin-embedded tissue sections were dewaxed using xylene and a graded series of ethanol. H&E staining was performed for microscopic analysis of mucosal damage using a previously described scoring system (2) **(Supplemental Table 1)**. Two blinded observers assessed histology scores using a Zeiss Axiostar Plus microscope in combination with an Axiocam MRC camera (Zeiss, Jena, Germany). For IHC, citrate-based antigen retrieval was performed (Dako Target Retrieval Solution, Agilent, Santa Clara, California, USA., #S1699) and sections were incubated overnight at 4°C with the following primary antibodies: PCNA (1:500, Abcam, Cambridge, UK, #265585), CC3 (1:100, Cell Signaling Technology (CST), Danvers, Massachusetts, USA, #9661), F4/80 (1:100, BioRad, Hercules, California, USA, #MCA497), CD3 (1:100, Abcam, #5690), pSTAT3 (1:100, CST, #9145), ERK1/2 (1:1000, CST, #9102), pERK1/2 (1:1000, CST, #9101). For IF on cryosections, a CD11c primary antibody was used (1:100, ThermoFisher Scientific, Waltham, Massachusetts, USA, #14-0114-82). After incubation with appropriate horse radish peroxidase or FITC (for IF-coupled secondary antibodies), detection was carried out using the Liquid DAB+ Substrate Chromogen System (Dako, #K3468). Quantification of positive cells was performed by two blinded investigators using a Zeiss Axiostar Plus microscope in combination with an Axiocam MRC camera (Zeiss).

qRT-PCR

RNA was isolated from murine colonic tumor and mucosa samples using the RNeasy Mini Kit (Qiagen, Hilden, Germany, #74104). cDNA was synthesized using the ImProm-II™ Reverse Transcription System (Promega, Mannheim, Germany, #A3800). qRT-PCR was performed on a LightCycler 480 system (Roche, Mannheim, Germany) using SYBR Green as a dye. Relative transcript expression was analyzed employing the ΔΔCt method with *18S rRNA (Rn18s)* or *actin beta* (*Actb*) as housekeeping genes. Primer sequences are listed in **Supplemental Table 2.**

Immunoblot

Protein from size- and location-matched AOM/DSS tumors was isolated using the AllPrep DNA/RNA/Protein Mini Kit (Qiagen, #80004). Isolated protein was quantified using the Pierce™ BCA Protein Assay Kit (ThermoFisher Scientific, #23225), and equal amounts were loaded for gel electrophoresis. Primary antibodies against STAT3 (1:1000, CST, #9139), pSTAT3 (1:1000, CST, #9145), ERK1/2 (1:1000, CST, #9102), pERK1/2 (1:1000, CST, #9101), ACTB (1:5000, Abcam, #8227) as well as an HRP-conjugated secondary antibody (1:5000, Abcam, #6721) were used for detection of the respective proteins.

Flow cytometry

The following antibodies were used to characterize lymphoid populations in AOM/DSS tumors: APC-R700-CD45 (30-F11) (BioLegend, 103128), Brilliant Violet 605-CD3 (17A2) (BioLegend, 100237), PE-CF594-CD4 (RM4-5) (BD, 562285), APC-C7, CD8a (53-6.7) (BioLegend, 100714), BB515-CD19 (1D3) (BD, 564531), Brilliant Violet 421-CD25 (PC61) (BD, 562606), APC-CD127, (SB/199), (BD, 564175), CD335/NKp46 (29A1.4) (BD, 560757).

For characterization of myeloid populations in AOM/DSS tumors, the following antibodies were used: APC-R700-CD45 (30-F11) (BioLegend, 103128), PE-Cy7-CD11b (M1/70) (BioLegend, 101216), Brilliant Violet 421-CD11c, (N418) (BioLegend, 117330), AF488-CD80 (16-10A1) (BioLegend, 104716), Brilliant Violet-786-CD86 (GL1) (BioLegend, 105043), PE-CF594-CD163 (S15049I) (BioLegend, 155316) PE -CD197/CCR7 (4B12) (BD, 560682), AF647-CD206 (MR5D3) (BD, 565250), APC-C7-F4/80 (BM8) (BioLegend, 123118), PerCP-Cy5.5-Ly-6C (HK1.4) (BioLegend, 128012) Brilliant Violet 605-Ly-6G (1A8) (BioLegend, 127639), BV510-MHC class II (M5/114.15.2) (BioLegend, 107636), BUV395-CD3 (17A2), (BD, 740268), BUV395-CD19 (1D3) (BD, 563557), BUV395-CD335/NKp46 (29A1.4) (BD, 740326). Incubation was performed in Brilliant Stain Buffer (BD, 563794). DAPI was added before data acquisition to identify viable cells.

Isolation of BMDMs

BMDMs were isolated as previously described (3). Briefly, femora and tibiae of WT and *Phd2*$^{+/-}$ mice were flushed with PBS, and bone marrow cells were differentiated to macrophages in RPMI-1640 medium supplied with 10% FCS, 1% Penicillin/Streptomycin, 2 mM L-Glutamine and 10 ng/ml murine M-CSF (R&D Systems, Minneapolis, Minnesota, USA, #416-ML) for seven days prior to experiments. For experiments, BMDMs were incubated for 24 hours with control media (RPMI-1640 medium supplied with 1% FCS), 100 ng/ml LPS in control media (Sigma-Aldrich, St. Louis, Missouri, USA, #L2630), 20 ng/ml TNFα in control media (R&D Systems, #410-MT), or 20 ng/ml IL-4 in control media (R&D Systems, #404-ML).

SUPPLEMENTAL FIGURES AND FIGURE LEGENDS

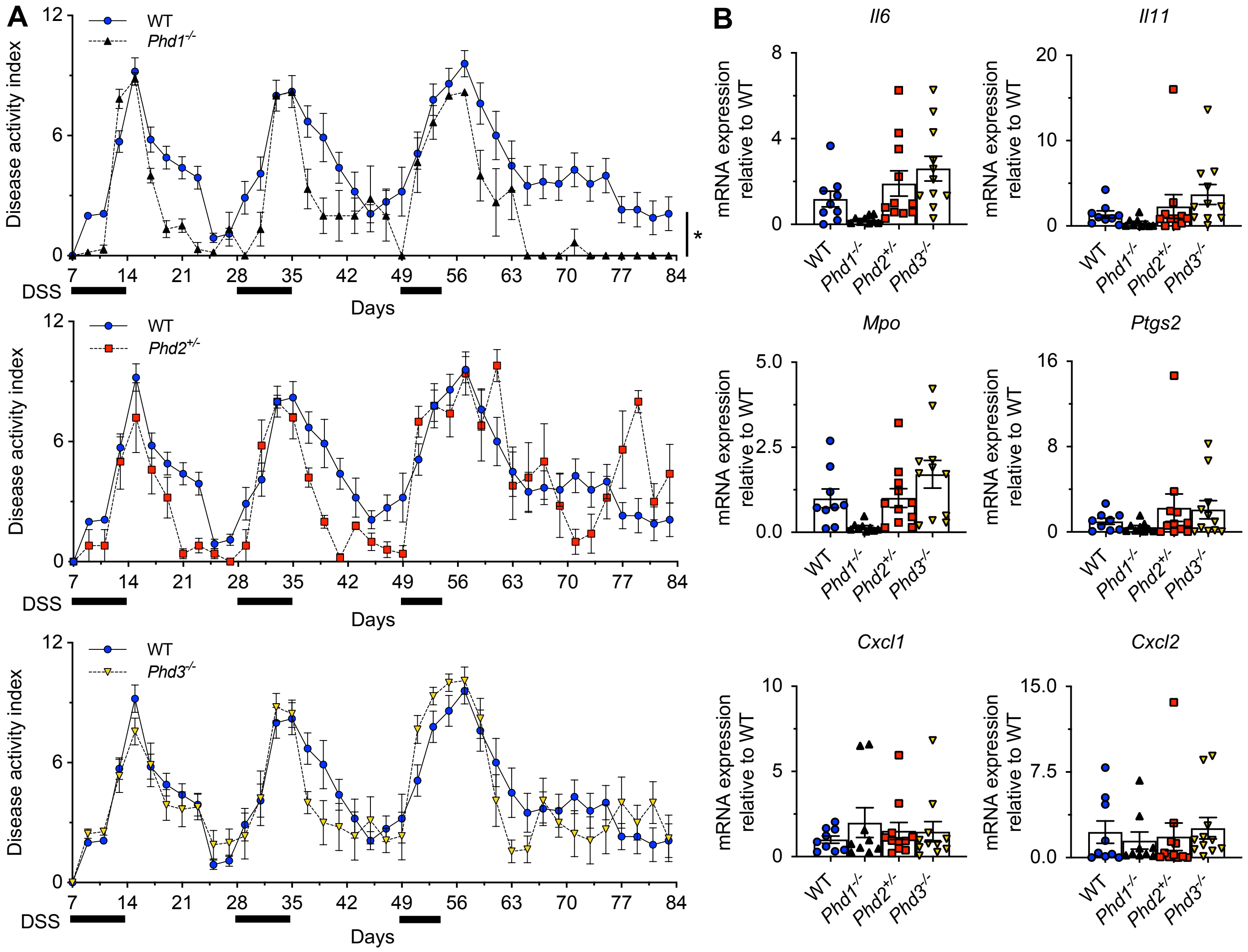

Supplemental Figure 1. (A) Disease activity index (DAI) scores from WT (n = 10), *Phd1*$^{-/-}$ (n = 6), *Phd2*$^{+/-}$ (n = 5), and *Phd3*$^{-/-}$ (n = 9) mice over the course of AOM/DSS treatment. The DAI was calculated every other day. (**B**) qRT-PCR analysis of pro-inflammatory mRNA expression in non-tumorous colon tissue samples from WT (n = 9), *Phd1*$^{-/-}$ (n = 9), *Phd2*$^{+/-}$ (n = 11), and *Phd3*$^{-/-}$ (n = 11) mice at day 84. Statistical significance was calculated using 2-way ANOVA (**A**) or 1-way ANOVA with Dunnett's multiple comparisons test (**B**). *P < 0.05.

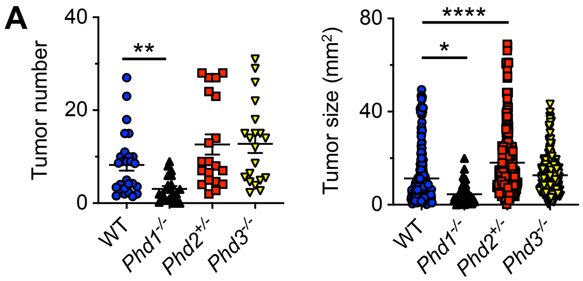

Supplemental Figure 2. (**A**) Macroscopic quantification of AOM/DSS-induced tumors. Pooled number of tumors per mouse (*left*; WT: n = 27, *Phd1*$^{-/-}$: n = 21, *Phd2*$^{+/-}$: n = 20, *Phd3*$^{-/-}$: n = 20 mice) and pooled size of individual tumors (*right*; WT: n = 220, *Phd1*$^{-/-}$: n = 63, *Phd2*$^{+/-}$: n = 255, *Phd3*$^{-/-}$: n = 243 tumors) of 4 studies in total. Statistical significance was calculated using 1-way ANOVA with Dunnett's multiple comparisons test. *P < 0.05, **P < 0.01, ****P < 0.0001.

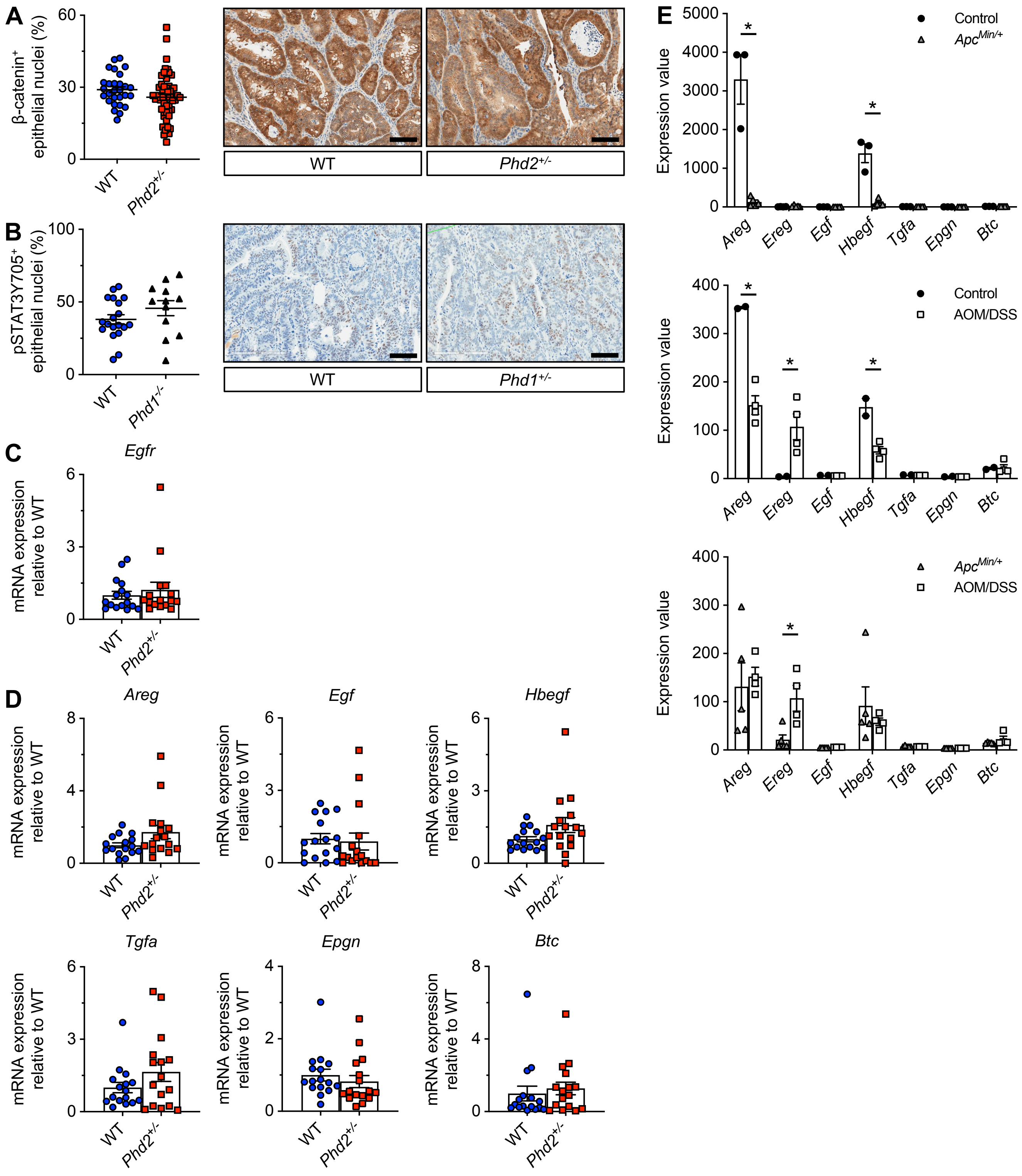

Supplemental Figure 3. (**A**) Quantification of epithelial nuclear β-catenin immunostaining in WT (n = 28) and *Phd2*$^{+/-}$ (n = 54) tumors and representative histological images. Scale bar = 100 μm. (**B**) Quantification of epithelial nuclear pSTAT3Y705 immunostaining in WT (n = 19) and *Phd1*$^{-/-}$ (n = 12) tumors and representative histological images. Scale bar = 100 μm. (**C, D**) qRT-PCR analysis of *Egfr* (**C**) and EGFR ligands mRNA expression (**D**) in WT (n = 16) and *Phd2*$^{+/-}$ (n = 16) tumors. (**E**) Re-analysis of a publicly available high-density microarray data set that includes transcriptomes from size- and location-matched AOM/DSS-induced and sporadic *Apc*$^{Min/+}$ tumors and respective controls (4). Statistical significance was calculated using 1-way ANOVA with Dunnett's multiple comparisons test (**A**) or Student's *t* test (**B - E**). *P < 0.05.

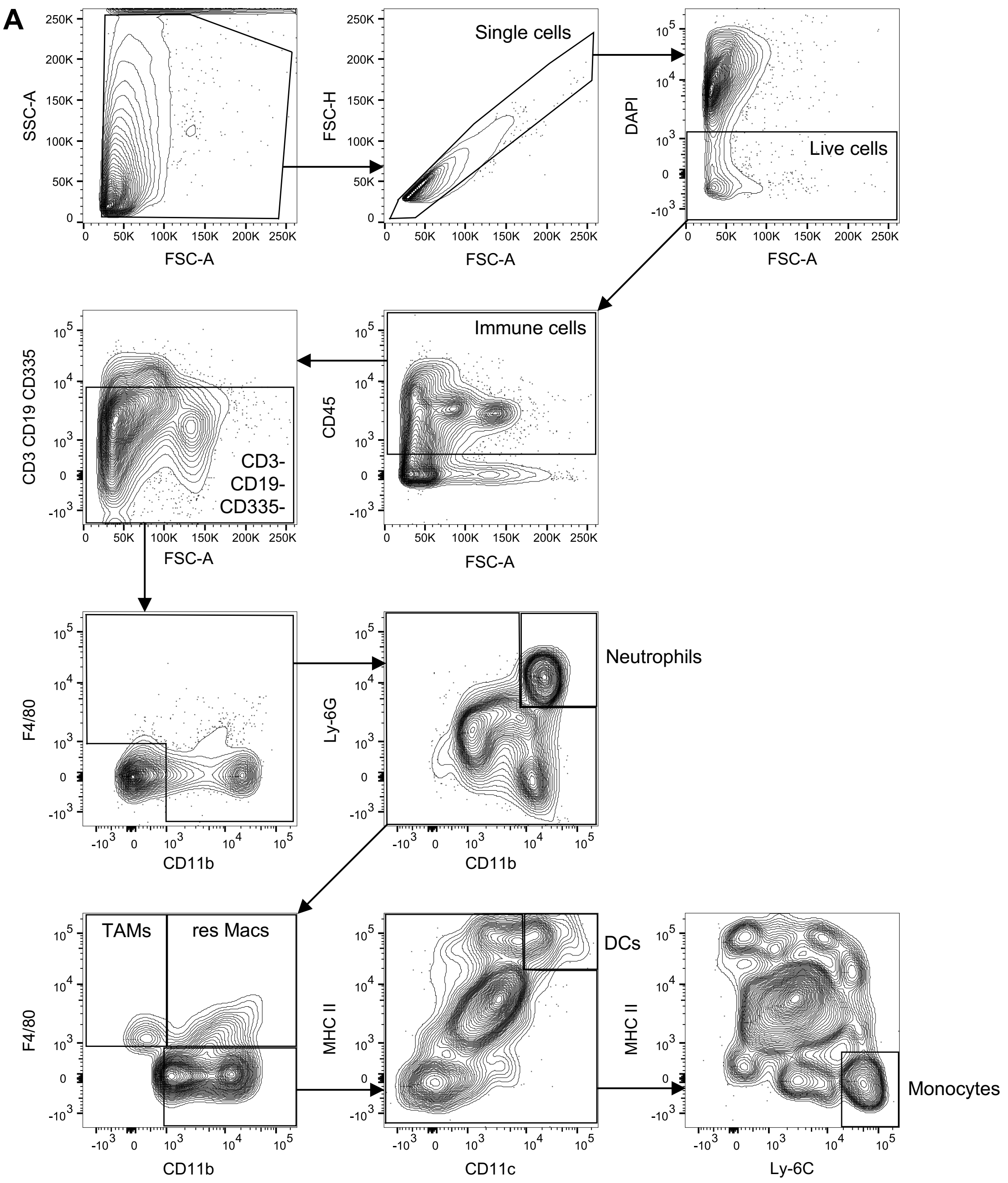

Supplemental Figure 4. (**A**) Flowcytometry gating strategy for myeloid populations in AOM/DSS tumors as previously established (5). First, single live cells were identified based on their FSC and SSC properties and DAPI staining. Next, immune cells (CD45+) were gated, and T, B, and NK cells excluded via CD3, CD19, and CD335 dump staining. Myeloid cells were then defined as neutrophils (CD45+, CD3-, CD19-, CD335-, Ly-6G+, CD11b+), Tumor-associated macrophages (TAMs, CD45+, CD3-, CD19-, CD335-, Ly-6G-, F4/80+, CD11b low), resident macrophages (res Macs, CD45+, CD3-, CD19-, CD335-, Ly-6G-, F4/80+, CD11b high), dendritic cells (DCs, CD45+, CD3-, CD19-, CD335-, Ly-6G-, F4/80-, CD11b+, MHCII+, CD11c+), monocytes (Mono, CD45+, CD3-, CD19-, CD335-, Ly-6G-, F4/80+, CD11b+, MHCII-, CD11c-, Ly-6C+). Representative contour plots from a *Phd2*$^{+/-}$ mouse.

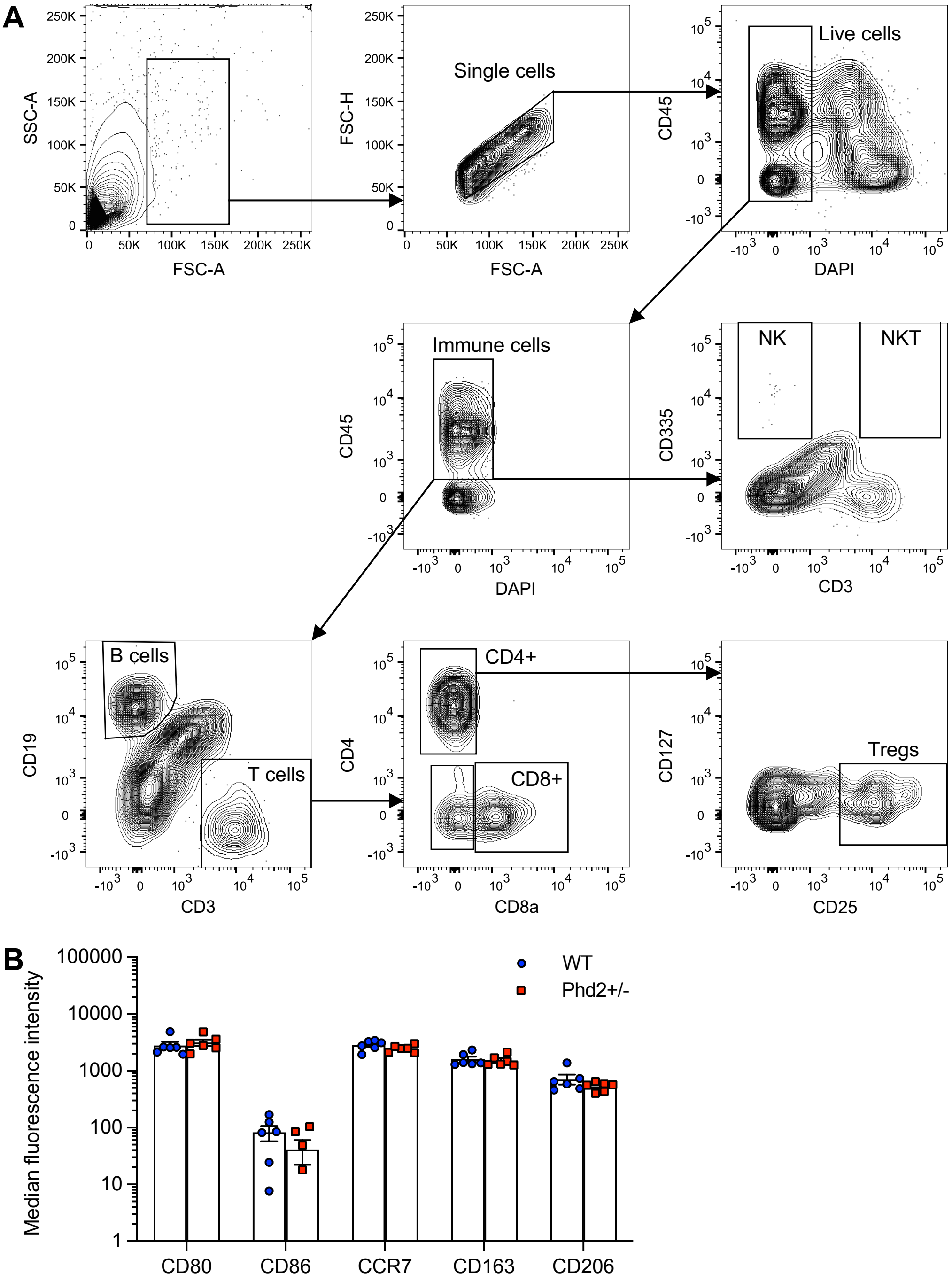

Supplemental Figure 5. (**A**) Flow cytometry gating strategy for lymphoid populations in AOM/DSS tumors. First, single live immune cells were identified based on their FSC and SSC properties, CD45, and DAPI staining. Next, lymphoid cells were defined as B cells (CD45+, CD19+), NK cells (CD45+, CD3-, CD335+), NKT cells (CD45+, CD3+, CD335+), T cells (CD45+, CD3+), Th cells (CD45+, CD3+, CD4+), regulatory T cells (Tregs, CD45+, CD3+, CD4+, CD127-, CD25+) and cytotoxic T cells (CD45+, CD3+, CD8+). Representative contour plots from a WT mouse. (**B**) Median fluorescence intensity for M1 (CD80, CD86, CCR7) and M2 (CD163, CD206) macrophage polarization markers within the TAM population in tumors from *Phd2*$^{+/-}$ (n = 6) and WT (n = 6) control mice. Statistical significance was calculated using Student‘s *t* test.

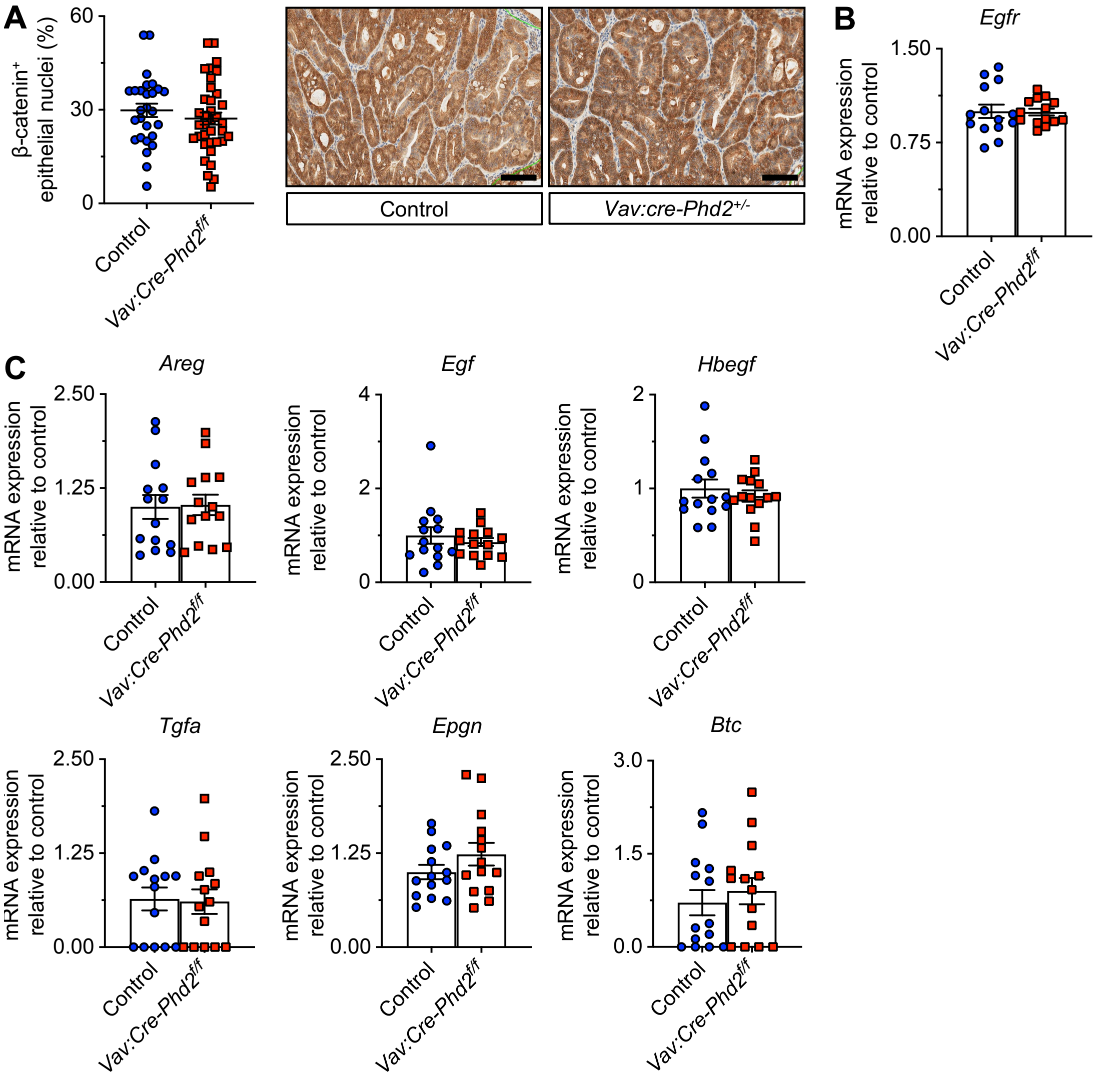

Supplemental Figure 6. (**A**) Quantification of epithelial nuclear β-catenin immunostaining in *Phd2*$^{f/f}$ (control, n = 27) and *Vav:Cre-Phd2*$^{f/f}$ mice (n = 36) and representative histological images (*right*). Scale bar = 100 μm. (**B-C**) qRT-PCR analysis of *Egfr* (**B**) and EGFR ligands (**C**) mRNA expression in control (n = 14) and *Vav:Cre-Phd2*$^{f/f}$ mice (n = 14).

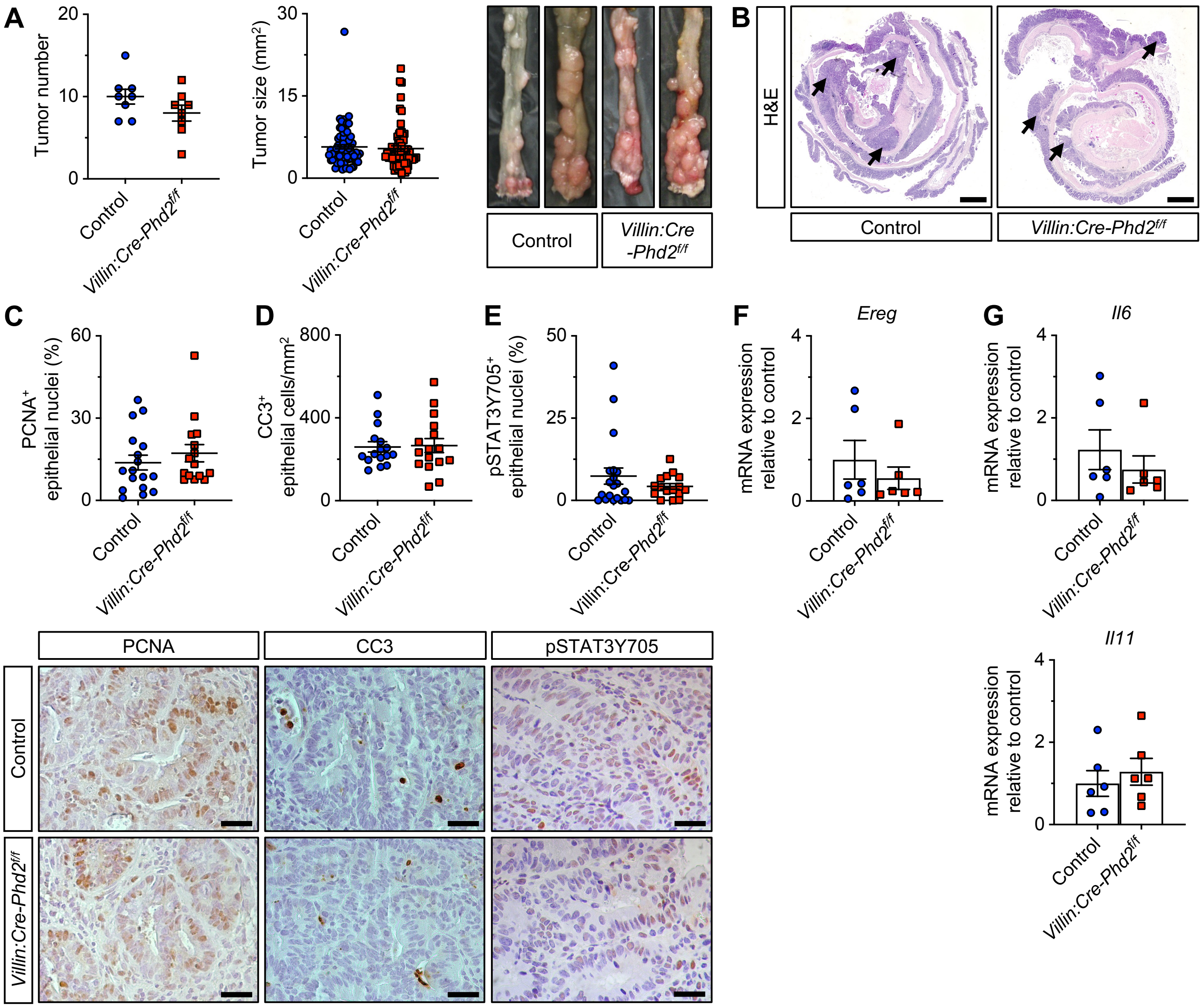

Supplemental Figure 7. (**A**) Macroscopic quantification of AOM/DSS-induced tumors in *Phd2*$^{f/f}$ (control) and *Villin:Cre-Phd2*$^{f/f}$ mice. Number of tumors per mouse (*left*; control: n = 8, *Villin:Cre-Phd2*$^{f/f}$: n = 8 mice) and size of individual tumors (*right*; control: n = 80, *Villin:Cre-Phd2*$^{f/f}$: n = 64 tumors). Representative macroscopic images of colons from control and *Villin:Cre-Phd2*$^{f/f}$ mice (*right*). **(B)** H&E stainings of colons from control and *Villin:Cre-Phd2*$^{f/f}$ mice. Arrows indicate colitis-associated tumors. Scale bar = 2 mm**. (C)** Quantification of epithelial PCNA immunostaining in control (n = 17) and *Villin:Cre-Phd2*$^{f/f}$ (n = 15) tumors and representative histological images (*bottom*). Scale bar = 25 µm. **(D)** Quantification of epithelial CC3 immunostaining in control (n = 16) and *Villin:Cre-Phd2*$^{f/f}$ (n = 16) tumors and representative histological images (*bottom*). Scale bar = 25 µm. **(E)** Quantification of epithelial nuclear pSTAT3Y705 immunostaining in control (n = 20) and *Villin:Cre-Phd2*$^{f/f}$ (n = 16) tumors and representative histological images (*bottom*). Scale bar = 25 µm. **(F-G)** qRT-PCR analysis of EGFR ligand *Ereg* (**F**) and *Il6* and *Il11* (**G**) mRNA expression in control (n = 6) and *Villin:Cre-Phd2*$^{f/f}$ (n = 6) tumors. Statistical significance was calculated using Student's *t* test.

Supplemental Tables

Score	Colonic epithelial damage	Inflammatory cell infiltration		Score
0	Normal epithelium	Mucosa	Normal	0
1	Hyperproliferation, irregular crypts, goblet cell loss		Mild	1
2	Mild to moderate crypt loss (10-50%)		Modest	2
3	Severe crypt loss (50-90%)		Severe	3
4	Complete crypt loss, surface epithelium intact	Submucosa	Normal	0
5	Small- to medium sized-ulcer (<10 crypt widths)		Mild to modest	1
6	Large ulcer (≥ 10 crypt widths)		Severe	2
		Muscle/serosa	Normal	0
			Moderate to severe	1

Supplemental Table 1. Histological scoring criteria for DSS-induced colitis. As previously described by Katakura et al. (2). Scores for epithelial damage and inflammatory cell infiltration (separately assessed for mucosa, submucosa. and muscle/serosa) are added, resulting in a minimum score of 0 and a maximum score of 12.

Supplemental Table 2. Primer sequences used for qRT-PCR

Gene	Primer type (FW, forward/REV, reverse)	Primer sequence
Il6	FW	TTCCTCTCTGCAAGAGACTTC
	REV	CTGTTGGGAGTGGTATCCTCTG
Il11	FW	GGGGACATGAACTGTGTTTGT
	REV	CAGGAGGGATCGGGTTAGGA
Mpo	FW	CTGCAACAACAGACGAAGCC
	REV	AGCCATTGCGATTGACTCCA
Ptgs2	FW	TCCCATGGGTGTGAAGGGAAA
	REV	ACCCAGGTCCTCGCTTATGA
Cxcl1	FW	ACCCAAACCGAAGTCATAGCC
	REV	TTGTCAGAAGCCAGCGTTCA
Cxcl2	FW	TCCAAAAGATACTGAACAAAGGCA
	REV	GCGAGGCACATCAGGTACG
Myc	FW	CTTTCCCTACCCGCTCAACG
	REV	CTTCTTGCTCTTCTTCAGAGTCG
Birc5	FW	GAACCCGATGACAACCCGAT
	REV	GTTGGTCTCCTTTGCAATTTTGT
Bcl2l1	FW	TGCGTGGAAAGCGTAGACAA
	REV	ACAAAAGTGTCCCAGCCGC
Ereg	FW	GACATGGACGGCTACTGCTT
	REV	TGTAGCCCACTTCACATCTGC
Rn18s	FW	GTAACCCGTTGAACCCCATT
	REV	CCATCCAATCGGTAGTAGCG
Actb	FW	TATAAAACCCGGCGGCGCA
	REV	TCATCCATGGCGAACTGGTG
Areg	FW	CTGAGGACAATGCAGGGTAA
	REV	AACCATCCGAAAGCTCCACT
Egf	FW	TTCTGGGTTCAGGACAGTGG
	REV	GACAAACTGTGCCGTGCTTG
Hbegf	FW	AGGACTTGGAAGGGACAGA
	REV	CCCATTCCTTTCTTTGCTTGG
Btc	FW	ATGGACCCAACAGCCCCGGGTAGCAGTGTC
	REV	TAACCGTTAAGCAATATTGGTCTCTTGAAT
Epgn	FW	GAGCGAAGAAGCAGAGGTGATC
	REV	GGTCTTCCAGACAAGGATGAGAG
Tgfa	FW	AGCCAGAAGAAGCAAGCCATCACT
	REV	TCATTCTCGGTGTGGGTTAGCAA
Egfr	FW	GAAGAAGTGCCCCCGAAACT
	REV	TCGTAGTAGTCAGGCCCACA

REFERENCES

1. Greten FR, Eckmann L, Greten TF, Park JM, Li ZW, Egan LJ, et al. IKKbeta links inflammation and tumorigenesis in a mouse model of colitis-associated cancer. *Cell.* 2004;118(3):285-96.
2. Katakura K, Lee J, Rachmilewitz D, Li G, Eckmann L, and Raz E. Toll-like receptor 9-induced type I IFN protects mice from experimental colitis. *J Clin Invest.* 2005;115(3):695-702.
3. Kiss J, Mollenhauer M, Walmsley SR, Kirchberg J, Radhakrishnan P, Niemietz T, et al. Loss of the oxygen sensor PHD3 enhances the innate immune response to abdominal sepsis. *J Immunol.* 2012;189(4):1955-65.
4. Neufert C, Becker C, Tureci O, Waldner MJ, Backert I, Floh K, et al. Tumor fibroblast-derived epiregulin promotes growth of colitis-associated neoplasms through ERK. *J Clin Invest.* 2013;123(4):1428-43.
5. Olesch C, Sirait-Fischer E, Berkefeld M, Fink AF, Susen RM, Ritter B, et al. S1PR4 ablation reduces tumor growth and improves chemotherapy via CD8+ T cell expansion. *J Clin Invest.* 2020;130(10):5461-76.
